# Ultrafast learning of four-node hybridization cycles in phylogenetic networks using algebraic invariants

**DOI:** 10.1093/bioadv/vbae014

**Published:** 2024-02-08

**Authors:** Zhaoxing Wu, Claudia Solís-Lemus

**Affiliations:** Department of Statistics, Wisconsin Institute for Discovery, University of Wisconsin-Madison, Madison, WI 53706, United States; Department of Plant Pathology, Wisconsin Institute for Discovery, University of Wisconsin-Madison, Madison, WI 53706, United States

## Abstract

**Motivation:**

The abundance of gene flow in the Tree of Life challenges the notion that evolution can be represented with a fully bifurcating process which cannot capture important biological realities like hybridization, introgression, or horizontal gene transfer. Coalescent-based network methods are increasingly popular, yet not scalable for big data, because they need to perform a heuristic search in the space of networks as well as numerical optimization that can be NP-hard. Here, we introduce a novel method to reconstruct phylogenetic networks based on algebraic invariants. While there is a long tradition of using algebraic invariants in phylogenetics, our work is the first to define phylogenetic invariants on concordance factors (frequencies of four-taxon splits in the input gene trees) to identify level-1 phylogenetic networks under the multispecies coalescent model.

**Results:**

Our novel hybrid detection methodology is optimization-free as it only requires the evaluation of polynomial equations, and as such, it bypasses the traversal of network space, yielding a computational speed at least 10 times faster than the fastest-to-date network methods. We illustrate our method’s performance on simulated and real data from the genus *Canis*.

**Availability and implementation:**

We present an open-source publicly available Julia package PhyloDiamond.jl available at https://github.com/solislemuslab/PhyloDiamond.jl with broad applicability within the evolutionary community.

## 1. Introduction

The Tree of Life is the graphical structure that represents the evolutionary process from single-cell organisms at the origin of life to present day biodiversity. Mathematically, a phylogenetic tree is a fully bifurcating graph in which its internal nodes represent speciation events that give rise to two children nodes. Recent evidence (Adavoudi and Pilot, 2021, De Santis *et al.* 2021, Pérez-Escobar *et al.* 2021, Rey *et al.* 2021, Suvorov *et al.* 2022) has challenged the notion that evolution across the Tree of Life can be represented with a fully bifurcating process, as this process cannot capture important biological realities like hybridization, introgression, or horizontal gene transfer that require two fully separated branches to join again. These processes of reticulate evolution are more prevalent in certain groups like plants (Charles and Stehlik, 2021), fungi (Steensels *et al.* 2021), and prokaryotes (Diop *et al.* 2022). To accurately include these groups in the Tree of Life, recent years have seen an explosion of methods to reconstruct phylogenetic networks, which naturally account for reticulate evolution (Degnan, 2018, Elworth *et al.* 2019, Blair and Ané, 2020, Kong *et al.* 2022). Existing methods, however, are still not scalable enough to tackle the complexities of the present day’s biological big data. The most scalable alternatives infer split (or implicit) networks (Bryant and Moulton, 2004, Huson and Bryant, 2006, Grünewald *et al.* 2007) which are not biologically interpretable as internal nodes no longer represent biological events (speciation or hybridization), and the resulting network is completely unrooted, even with the inclusion of an outgroup.

Among methods to infer explicit networks (those whose internal nodes do represent speciation or hybridization events), likelihood-based approaches are among the most popular ones. PhyloNet (Yu *et al.* 2014) has been the pioneer of likelihood inference of phylogenetic networks, later expanding to Bayesian (Wen *et al.* 2016) and pseudolikelihood alternatives (Yu and Nakhleh 2015). However, even in its most scalable option (pseudolikelihood), the method is still not suitable beyond dozens of taxa and hundreds of genes. SNaQ (Solís-Lemus and Ané 2016) within the PhyloNetworks Julia package (Solís-Lemus *et al.* 2017) has proven a scalable alternative to infer large phylogenetic networks from multilocus alignments. This method is based on a pseudolikelihood model under the coalescent, and its running time does not increase as a function of number of genes because the input data for SNaQ are split frequencies on subsets of four taxa (concordance factors). That is, after the estimation of the concordance factors, the running time is the same for data with 10 genes or 10 000 genes. Despite its popularity, SNaQ still lacks scalability as the number of taxa increases and remains a suitable alternative only for 50 or fewer taxa. Last, BEAST2 (Zhang *et al.* 2018) provides the only co-estimation method that infers simultaneously the phylogenetic networks and the gene trees, and it allows users to infer a variety of relevant biological parameters (such as divergence times), yet the complexity of this method renders it unsuitable for anything beyond a handful of taxa.

Because of the complexity in inferring a phylogenetic network, the evolutionary biology community has embraced the use of hybrid detection methods such as ABBA-BABA test (Patterson *et al.* 2012), MSCquartets (Mitchell *et al.* 2019, Rhodes *et al.* 2021), and HyDe (Kubatko and Chifman 2019) to identify hybridization events on a fixed phylogeny. These methods take in subsets of taxa and test whether the current subset can be well-explained with a tree-like pattern or if a hybridization event exists in this subset. While fast, these methods can be inaccurate in the presence of multiple hybridization events affecting the same taxa (Bjorner *et al.* 2022) or by ghost lineages (Bjorner *et al.* 2022, Pang and Zhang 2023, Tricou *et al.* 2022). Furthermore, the process of first reconstructing a phylogenetic tree and then adding hybridization events can be flawed given the bias that gene flow causes on the inference of the backbone phylogenetic tree (Solís-Lemus *et al.* 2016).

Here, we introduce a novel method to infer hybridization events using phylogenetic invariants. Our method only requires evaluation of polynomial equations, so it bypasses optimization in the space of networks and it is at least 10 times faster than current inference network methodologies. Using gene trees as input, our method exploits the signal in the frequencies of splits in the data, also well-known as *concordance factors*. Indeed, under the coalescent model, splits of taxa display certain probabilities of appearing in the sample of gene trees. Under the true network, these frequencies need to satisfy certain polynomial equations, denoted *phylogenetic invariants*. By plugging in the observed frequencies on the phylogenetic invariants of every possible placement of the hybridization cycle, we can identify the hybridization cycle that better agrees with the data as the one whose evaluated invariants are closest to zero.

Algebraic invariants have been widely used in phylogenetics to identify trees under a variety of models of evolution (Jukes-Cantor (Felsenstein 1991, Steel and Fu 1995), GTR (Allman and Rhodes 2003, Fernández-Sánchez and Casanellas 2016, Casanellas *et al.* 2021, 2023)), and more recently, to identify phylogenetic networks (Gross and Long 2018, Gross *et al.* 2021, Ardiyansyah 2021, Cummings *et al.* 2024, Barton *et al.* 2022). Nevertheless, prior work on phylogenetic invariants in networks has been mainly focused in identifying polynomial relationships on the parameters in models of evolution. For example, Gross and Long (2018) identify relationships among site pattern frequencies that need to hold for certain network parameters under Jukes Cantor model of evolution. In contrast, our work is the first to identify polynomial relationships (phylogenetic invariants) on concordance factors within the framework of the multispecies coalescent model on networks.

This work first introduces the phylogenetic invariants that concordance factors need to satisfy under level-1 phylogenetic networks with a four-node hybridization cycle under the multispecies coalescent model. Next, we describe an inference methodology to use the phylogenetic invariants to infer the correct four-node hybridization cycle among *n* taxa. Moreover, we demonstrate the performance of our method with simulated data, and we revisit the inference of the *Canis* phylogenetic network that had already been published in Gopalakrishnan *et al.* (2018). We show that our method is faster than all existing network inference methods, and it accurately identifies the correct hybridization cycle in the *Canis* genus. Additionally, in all simulating scenarios, our method recognizes the correct placement of hybridization events with high accuracy. Even in the few cases when our method identifies an incorrect network (usually cases when there is not sufficient sampling of taxa that descend from the hybrid node), the correct network is still ranked among top five optimal networks given the invariant score (evaluated phylogenetic invariants that need to vanish to zero). This means that, regardless of sampling, our method is always able to reduce the space of candidate networks that can later be tested with a likelihood-based approach.

We highlight that our method has two main limitations: (i) it is only suitable to identify level-1 hybridization cycles with four nodes, and (ii) it sometimes fails to identify the correct network as the one with the top 1 ranked invariant score when there is only one taxon sampled that is descendant of the hybrid node. Despite these limitations, our method is a valuable innovation in the landscape of phylogenetic network methods, if anything, to reduce the space of candidate networks to be used in likelihood-based methodologies. Indeed, we do not consider our method as a replacement for other network inference approaches. On the contrary, we believe that our ultrafast learning methodology can serve to (i) identify the true phylogenetic network when it involves a simple hybridization event, and (ii) provide several candidate networks to be later tested with a likelihood-based approach, effectively bypassing heuristic optimization in the space of networks. Furthermore, our method is open source, publicly available as the new Julia package PhyloDiamond.jl in https://github.com/solislemuslab/PhyloDiamond.jl.

The structure of the article is as follows. In Section 2, we introduce the phylogenetic invariants under the multispecies coalescent model on networks, and we describe an inference methodology that uses the phylogenetic invariants to identify the four-node hybridization cycle that generates the data. In this section, we also depict the simulation study and the re-analysis of the *Canis* data from Gopalakrishnan *et al.* (2018). In Section 3, we present the results on the simulated data and the *Canis* phylogeny. Last, in Section 4, we explain the main limitations of our method and potential future work.

## 2. Materials and methods

### 2.1 Phylogenetic invariants for four-node hybridization cycles in level-1 phylogenetic networks

Under the coalescent model, the distribution of gene trees estimated from multilocus sequence alignments provides information on the true network that generated the data (Yu *et al.* 2014). In Solís-Lemus and Ané (2016), it was shown that the split frequencies on subsets of four taxa, namely the *concordance factors* (CF), also provide information on the true network. That is, a CF of a given quartet (or split) is the proportion of genes whose true tree displays that quartet (or split) (Baum 2007). For example, for a taxon set s={a,b,c,d}, there are only three possible quartets, represented by the splits $q_{1}=ab|cd$, $q_{2}=ac|bd$, and $q_{3}=ad|bc$. The CF for the split ab|cd is the proportion of gene trees that display this split.

**Figure 1.**
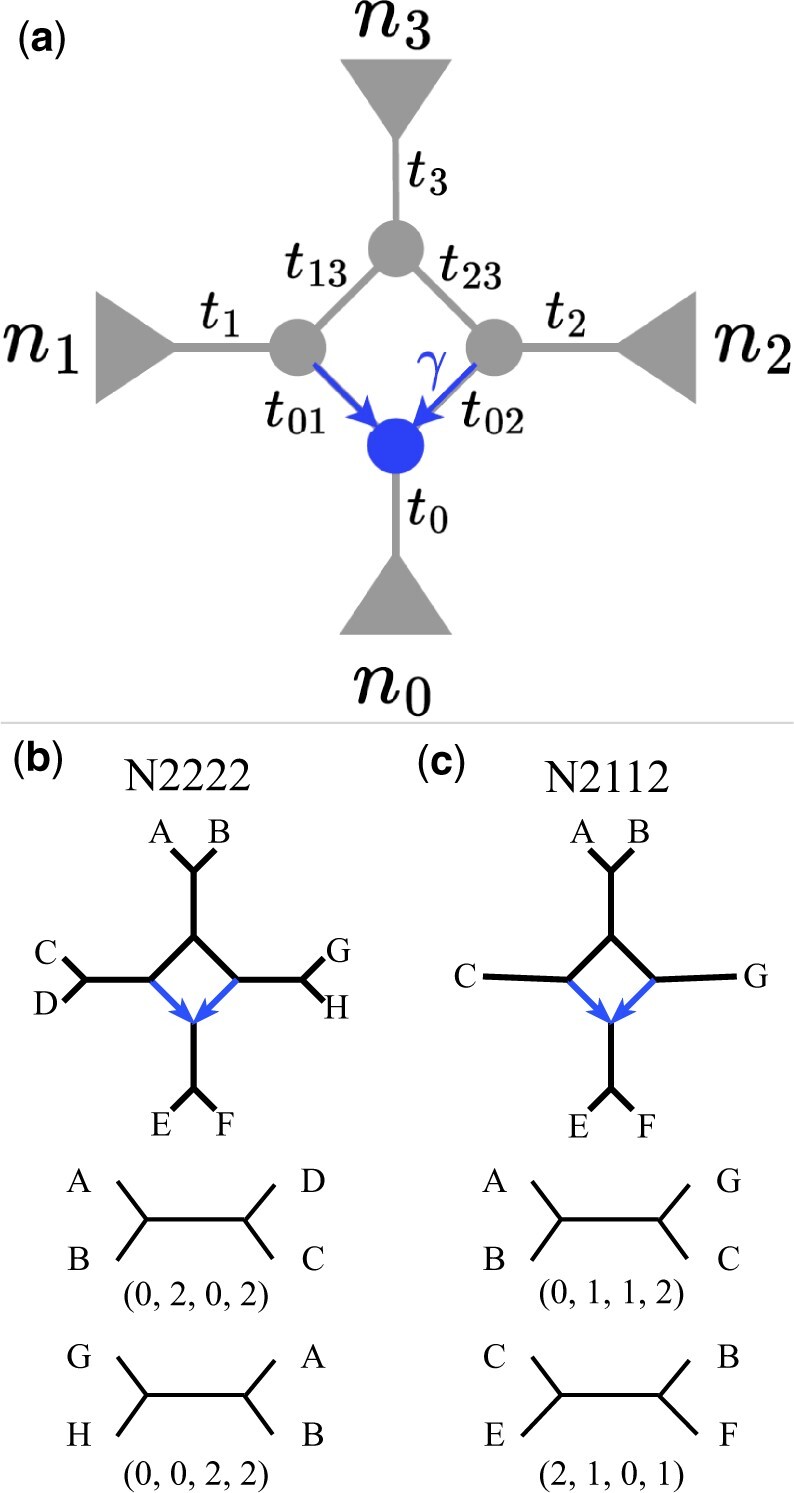
(a) Four-node hybridization cycle on a level-1 phylogenetic network where $t_{i}$ represents the branch length in coalescent units. The hybrid node and hybrid edges are in blue. The minor hybrid edge is labeled with γ that represents the inheritance probability (or proportion of genes transferred through the minor edge). Denote this level-1 semi-directed network with four-node hybridization cycles as *N*. (b) An example of a level-1 eight-taxon network with one four-node hybridization cycle and two examples of quartets drawn from the network above along with their vector notations. For example, the quartet AB|CD drawn from the N2222 network corresponds to the four-taxon subset (0,2,0,2) where each entry in this vector corresponds to the number of taxa drawn from each of the four clades: $n_{0},n_{1},n_{2},n_{3}$. (c) Another example of a level-1 six-taxon network with one four-node hybridization cycle and two examples of quartets drawn from the network above along with their vector notations.

As in Solís-Lemus and Ané (2016), our method uses the CFs as input data, and we focus on the case of four-node hybridization cycles on level-1 phylogenetic networks as shown in Fig. 1a. A level-1 phylogenetic network is a network with no vertex belonging to more than one hybridization cycle. The semi-directed network in Fig. 1a is unrooted, yet the direction of the hybrid edges (in blue) is known so that the root placement of the network is constrained. Namely, the root cannot be anywhere below the hybrid node (in blue). The hybridization cycle partitions the taxa into four subsets with $n_{0},n_{1},n_{2}$ and $n_{3}$ as the number of taxa in each subset. For example, the clade below the hybrid node in blue in Fig. 1a, also known as the hybrid clade, has $n_{0}$ taxa, and the two sister clades to the hybrid clade have $n_{1}$ and $n_{2}$ taxa. The notation $n_{i}$ not only represents the number of taxa in a specific clade, this terminology is also used to refer to that specific clade. In fact, we refer to the hybrid clade as the $n_{0}$ clade. This $n_{i}$ notation will later help us represent different network structures and four-taxon subsets. Moreover, each clade in the network could be a subtree or a subnetwork as long as the overall network is level-1 (Huson *et al.* 2010). Last, we highlight that by “taxa,” we mean either different species or populations, or multiple individuals sampled from the same species.

In this work, we introduce a method to infer four-node hybridization cycles in level-1 semi-directed networks. Since we focus on level-1 four-node hybridization cycles (as in Fig. 1a), we utilize a simplifying notation to represent each network. Let *N* represent an n−taxon semi-directed level-1 phylogenetic network, and we represent this network by the number of taxa in its four clades. For example, in Fig. 1b, the network denoted as N2222 has two taxa in each of the four clades, while the network N2112 in Fig. 1c contains two taxa in clades $n_{0}$ and $n_{3}$, and one taxon in clades $n_{1}$ and $n_{2}$.

Another relevant notation is the vector representation of every four-taxon subset. For example, in Fig. 1b, the four-taxon subset (0,2,0,2) drawn from network N2222 corresponds to the case when two taxa are taken from $n_{1}$ and $n_{3}$ no taxon is taken from $n_{0}$ and $n_{2}$, and this subset corresponds to the quartet AB|CD. Another example is the four-taxon subset (0,1,1,2) drawn from network N2112 in Fig. 1c. Note that since this network only has one taxon in clades $n_{1}$ and $n_{2}$, any four-taxon subset drawn from this network must have at most one taxon in the second and third value in the four-taxon vector notation. In general, a four-taxon subset is represented by a four-dimensional vector so that each element in this vector corresponds to the number of taxa drawn from each of the four clades $n_{0},n_{1},n_{2},n_{3}$.

Next, we describe the formulas for the expected CFs under the coalescent model for every four-taxon subset on a level-1 semi-directed network with branch lengths in coalescent units ($t_{i}$) and inheritance probability (γ). These formulas have already been derived in Solís-Lemus and Ané (2016). Let *N* be the network on Fig. 1a, and let (0,0,2,2) be the four-taxon subset (see Fig. 1b for an example) for which we want to compute the CF formulas under the coalescent model. Let taxa $k_{1},k_{2}\in n_{2}$ and taxa $l_{1},l_{2}\in n_{3}$. The probability of taxa $k_{1}$ and $k_{2}$ coalescing in a branch with length $t_{2}\;+\;t_{23}\;+\;t_{3}$ is given by $1-\frac{2}{3}z_{2}z_{23}z_{3}$ where $z_{i}=\text{exp}(-t_{i})$. Then, the formulas for the three CF under the coalescent model are defined as:

(1) $$\begin{array}{c} P(k_{1},k_{2}|l_{1},l_{2})=1-\frac{2}{3}z_{2}z_{23}z_{3}=a_{1} \end{array},$$

(2) $$P(k_{1},l_{1}|k_{2},l_{2})=\frac{1}{3}z_{2}z_{23}z_{3}=a_{2},$$

(3) $$\begin{array}{c} P(k_{1},l_{2}|k_{2},l_{1})=\frac{1}{3}z_{2}z_{23}z_{3}=a_{3} \end{array},$$

where $a_{1},a_{2}$, and $a_{3}$ denote the values for the true CFs corresponding to the splits $k_{1},k_{2}|l_{1},l_{2}$, $k_{1},l_{1}|k_{2},l_{2}$, and $k_{1},l_{2}|k_{2},l_{1}$, respectively. Thus, for the four-taxon subset (0,0,2,2), there are three polynomial equations that represent the probabilities for each quartet under the coalescent model.

In Supplementary Material, we list all of the CF formulas for all four-taxon subsets on a level-1 semi-directed network with one four-node hybridization cycle (Fig. 1a). Because of the theoretical work in Solís-Lemus and Ané (2016), Solís-Lemus *et al.* (2020), we know that we only need two taxa per clade to represent all CF formulas involving the hybridization cycle, and thus, we can restrict to the case of n=8 taxa for the definition of all CF formulas (yet our method is not restricted to eight taxa, see Inference of a four-node hybridization cycle in an *n*-taxon phylogenetic networks using phylogenetic invariants). Furthermore, not all $\left(\begin{array}{c} 8\\ 4 \end{array}\right)=70$ four-taxon subsets are required. Indeed, we ignore four-taxon subsets of the form (3,1,0,0) since three or more taxa from one of the clades (in this case, three taxa from clade $n_{0}$) do not provide information about the hybridization cycle. Recall that only the length of internal branches appears in the CF formulas but not the length of external branches. Therefore, if there are three or more taxa on one clade (e.g. three taxa in clade $n_{0}$), then branches involved in the hybridization cycle will correspond to an external branch and thus, will not be included in any of the CF formulas. The same is true if all four taxa in the four-taxon subset all come from the same clade. Thus, only four-taxon subsets where at most two taxa come from a given clade contain information about the hybridization cycle. There are 19 such 4-taxon subsets in which 0≤i,j,k,l≤2 for (i,j,k,l), and thus, there are 19×3=57 concordance factor polynomial equations along with 57 true CF values ($a_{i}$).

As just described, the semi-directed network *N* in Fig. 1a can be represented by a set of 57 polynomial equations, denoted CF(N) in 66 variables: 9 variables corresponding to branch lengths and inheritance probability ($z_{0},z_{1},z_{2},z_{3},z_{01},z_{02},z_{13},z_{23},\gamma$ for $z_{i}=\text{exp}(-t_{i})$) and 57 variables corresponding to the true CF values ($a_{i}$ for i=1,…,57). Here, we identify the relationships that the $a_{i}$ values need to satisfy for the polynomial system CF(N) to be consistent. These relationships are denoted *phylogenetic invariants*. For example, because the three concordance factors corresponding to a given four-taxon subset (say (0,0,2,2) as described above) need to sum up to one, we have one phylogenetic invariant for these three numbers: $a_{1}+a_{2}+a_{3}=1$, or similarly $i_{1}(a_{1},a_{2},a_{3})=a_{1}+a_{2}+a_{3}-1$ with $i_{1}(a_{1},a_{2},a_{3})=0$. In addition, since the two minor concordance factors are equal, we have a second phylogenetic invariant defined for these numbers: $a_{2}=a_{3}$, or similarly $i_{2}(a_{2},a_{3})=a_{2}-a_{3}$ with $i_{2}(a_{2},a_{3})=0$. It turns out that the set of all concordance factor values for all four-taxon subsets (the 57 polynomial equations denoted CF(N)) define a set of phylogenetic invariants (equations only in the $a_{i}$ variables) that need to vanish to zero whenever the concordance factors come from the true network. In this way, we can evaluate whether the given CF values come from the true network or not without knowing ($t_{i}$) and (γ).

Note that not all networks will have the same number of CF polynomial equations. For example, for the eight-taxon network N=2222, all 19 four-taxon subsets can be extracted, and thus, all 57 CF equations are defined in CF(N). For the five-taxon network N=1112, the four-taxon subset (2,1,1,0) cannot be considered since this subset requires two taxa from the clade $n_{0}$ and the five-taxon network only has one taxon in this clade. Therefore, the five-taxon network N=1112 defines a set of fewer polynomial equations (12 to be exact) in CF(N) involving only the CF values: $a_{7},a_{8},a_{9},a_{22},a_{23},a_{24},a_{28},a_{29},a_{30},a_{31},a_{32}$, and $a_{33}$.

To obtain the phylogenetic invariants defined by *N*, denoted I(N), we get the Gröbner basis of CF(N) on the $a_{i}$ variables using any elimination method in Macaulay2 (Grayson and Stillman). All Macaulay2 scripts (and output files) are publicly available in https://github.com/solislemuslab/PhyloDiamond.jl.

For example, for the network N=1112, there are 10 phylogenetic invariants in the Gröbner basis of CF(N) on the $a_{i}$ variables:

1. $a_{32}-a_{33}$
2. $a_{31}+2a_{33}-1$
3. $a_{28}+a_{29}+a_{30}-1$
4. $a_{23}-a_{24}$
5. $a_{22}+2a_{24}-1$
6. $a_{8}-a_{9}$
7. $a_{7}+2a_{9}-1$
8. $3a_{9}*a_{30}+a_{9}-a_{24}-a_{33}$
9. $a_{24}*a_{29}+2a_{24}*a_{30}+a_{29}*a_{33}-a_{30}*a_{33}-a_{33}$
10. $3a_{9}*a_{29}-2a_{9}+2a_{24}-a_{33}$

We consider the first seven invariants as the “trivial” invariants related to the sum-to-one property and the equality of the minor CFs. The sum-to-one property needs to be satisfied by the three CFs for any given quartet, and while the equality of the minor CFs is not necessarily true for all quartets, it is a simpler invariant compared to the other polynomial equations. The last three invariants, on the other hand, identify relationships that the true CFs need to satisfy if they originated from the N=1112 network. We note that the phylogenetic invariants depend on CF values $a_{i}$ but neither on branch lengths $t_{i}$ nor on inheritance probabilities γ. This property is desirable because when inferring a network, CFs values $a_{i}$ can be estimated from gene trees or genetic sequences, but both *t* and γ are unknown parameters. All the phylogenetic invariants corresponding to each n−taxon network (5≤n≤8) can be found in the Supplementary Material.

### 2.2 Inference of a four-node hybridization cycle in an *n*-taxon phylogenetic networks using phylogenetic invariants

**Figure 2.**
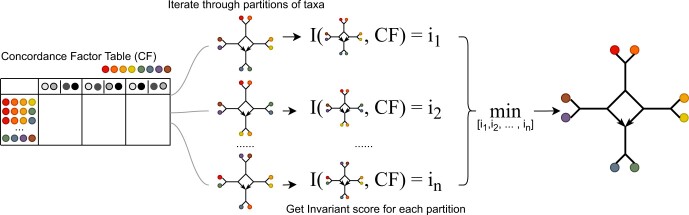
Schematic of the methodology using phylogenetic invariants to identify the best placement of the four-node hybridization cycle to fit the table of observed concordance factors. In this case, there are eight taxa shown as colored circles. The invariant score is defined as the $L_{2}$ norm of the vector of evaluated invariants.

The procedure to infer a phylogenetic network with one four-node hybridization cycle using phylogenetic invariants starts with a table of estimated CFs. As shown on Fig. 2 (left), the CF table contains rows representing different four-taxon subsets and three columns labeled by grey-scale circles denoting three types of bipartitions. Each value in this table is mapped to the vector of $\left[\begin{array}{ccc} a_{1} & \ldots & a_{57} \end{array}\right]$ and this vector is plugged in the invariants for each candidate network I(N). Note that the invariants are obtained on the true CFs values $\left(a_{1},\ldots ,a_{57}\right)$ which are subsequently estimated with the observed CFs from data. Here, all candidate networks are all possible partitions of given taxa. For example, as mentioned, the candidate network N=1112 has 10 phylogenetic invariants (|I(N)|=10), and thus, after taking in the observed CFs, the invariants output a 10-dimensional vector. The *invariant score* is defined as the $L_{2}$ norm of the vector of evaluated invariants and the candidate network with the smallest invariant score is identified as the network that agrees best with the observed CFs. See Fig. 2 for a graphical abstract of the procedure and Algorithm 1 for a more detailed description of the steps.

Algorithm 1 Inference of a four-node hybridization cycle in an *n*-taxon phylogenetic network (5≤n≤8) with phylogenetic invariants

**Input:** Table of estimated concordance factors; optional: the number of optimal networks (*m*) to return (default m=5)

**Output:** Top *m* optimal networks with smallest invariant score

scores ← an empty array

nets ← an empty array

**for** $N_{i}$ *, a possible partition of taxa* **do**

  append(nets,$N_{i}$)

  map observed CF values to $a_{i}$ values

  score ← L2norm($I(N_{i})$)

  append(scores, score)

**end**

rank(scores, nets)

**return** scores[1: m], nets[1: m]

Given that we only have phylogenetic invariants for the cases of 5≤n≤8 taxa, if the true network has 5≤n≤8 taxa, the procedure is just as described in Fig. 2 and Algorithm 1. If the true network has more than eight taxa, then the procedure is slightly different. In this case, our method needs to iterate through all possible subsets of eight taxa, and fit the phylogenetic invariants on each subset to identify the top subnetwork with n=8 taxa. Let $N_{8}^{*}$ be the best eight-taxon network to fit the observed CFs. The missing taxa are added to $N_{8}^{*}$ according their placement in the second best network, or if the missing taxa are not in the second best network, the algorithm adds them according to their placement in the third best or the fourth best and so on. For example, assume that we have n=9 taxa (A, B, C, D, E, F, G, H, I) and the best network with n=8 taxa ($N_{8}^{*}$) contains A, B, C, D, E, F, G, H in the following partition: A, B in $n_{0}$; C, D in $n_{1}$; E, F in $n_{2}$, and G, H in $n_{3}$. We then find the best network containing taxon I. Assume that this network puts taxon I in the clade $n_{2}$, then the resulting best network would have E, F, and I in $n_{2}$. Note that our algorithm is unable to resolve the bifurcation structure within a given clade (e.g. the $n_{2}$ clade here with E, F, and I), but it is able to (i) correctly identify the partition of taxa among the four clades defined by the four-node hybridization cycle and (ii) correctly identify those are in the hybrid clade ($n_{0}$), those are in the sister clades to the hybrid clade ($n_{1}$ and $n_{2}$), and those are in the remaining fourth clade ($n_{3}$). See Algorithm in the Supplementary Appendix for the steps in the procedure for n>8 taxa.

### 2.3 Simulation study

#### 2.3.1 Networks with eight or fewer taxa

In this section, we focus on the case of six taxa (N=2211,2121,2112,1122,1212,1221), seven taxa (N=2221,2212,2122,1222) and eight taxa (N=2222). Next subsection describes the simulations with more than eight taxa.

For a given network *N*, we generate data under four settings: (i) true concordance factors (proof of concept); (ii) true concordance factors perturbed by Gaussian error; (iii) simulated gene trees, and (iv) estimated gene trees. To generate the true concordance factors on a given network in the first scenario, we use the Julia package PhyloNetworks.jl (Solís-Lemus *et al.* 2017), and this scenario is designed to demonstrate that the invariants method works under ideal conditions. For the second setting, we add Gaussian noise to the true concordance factors with zero mean and standard deviation of σ=0.0005,0.00005,0.000005. We choose Gaussian noise as is standard practice. In the third setting, we use ms-converter that runs ms (Hudson 2002) to simulate k=100,1000,10000 gene trees under the coalescent model on a given network *N*. All internal branches in the network are set to 1.0 coalescent unit so that the amount of incomplete lineage sorting is controlled, but not trivial, and we set an inheritance probability parameter of γ=0.3. For the fourth setting, we simulate sequences of length L=500,2000 bp on each simulated gene tree with seq-gen (Rambaut and Grass 1997) under the HKY model, scale the branch lengths by 0.036, and set nucleotide frequencies as 0.300414, 0.191363, 0.196748, and 0.311475. Then, we estimate gene trees with IQ-Tree (Minh *et al.* 2020) with ModelFinder Plus for model selection (Kalyaanamoorthy*et al.* 2017). We repeat each simulation setting 30 times. All simulation scripts are in the GitHub repository https://github.com/solislemuslab/PhyloDiamond.jl.

#### 2.3.2 Networks with more than eight taxa

We also analyze cases of nine taxa (N=2223,2232,2322,3222) and ten taxa (N=3322,3232,3223,2233,2323,2332). Given that the algorithm to infer a network with more than eight taxa relies entirely on the algorithm on eight taxa, we focus on extensive simulations for the algorithm on eight taxa in the previous section, and simply show proof-of-concept simulations for the case of more than eight taxa in this section. In particular, we focus on testing the following scenarios: (i) true concordance factors, and (ii) true concordance factors perturbed by Gaussian error as described above (σ=0.0005).

#### 2.3.3 Reticulate evolution in the genus *Canis*

We further test our method on the *Canis* dataset from Gopalakrishnan *et al.* (2018). The original genomic dataset contained 12 gray wolves, 14 dogs, five coyotes, one Ethiopian wolf, three golden jackals, six African golden wolves, two dholes, four African hunting dogs, and one Andean fox. Given the widespread gene flow reported in the original study (Gopalakrishnan *et al.* 2018), we need to subsample the taxa so that (i) there is one four-node hybridization event and (ii) the phylogeny is fully resolved and do not have any ambiguous relationships within clades. Eventually, the selected taxa contain one African hunting dog, one coyote, one dhole, one dog, one golden jackal, and one grey wolf. We use the estimated gene trees from Zhang and Mirarab (2022) available in https://github.com/chaoszhang/Weighted-ASTRAL_data to infer the concordance factors with the Julia package PhyloNetworks (Solís-Lemus *et al.* 2017). We note that it is not necessary to subsample the taxa to use our method. The algorithm described for more than eight taxa is capable of identifying the four-node hybridization cycle for any number of taxa. However, as mentioned, its limitation is that we are unable to resolved the relationships within the four clades. In this case, we decided to subsample the species in order to obtain a fully resolved phylogeny.

To better evaluate our method’s performance, we also run other methods on this dataset for comparison. In addition to our method, we also run SNaQ (Solís-Lemus and Ané 2016) on h=1 hybridization event, 10 independent runs, and using one randomly selected gene tree as the starting tree. We use all 449 450 gene trees to infer the table of CFs and then map the allele names to species names. We also run PhyloNet on two options: (i) maximum likelihood (ML) and (ii) maximum pseudolikelihood (MPL). We choose h=1 hybridization event on both cases, with 10 independent runs for each. We choose one representative individual per species in the gene trees and removed gene trees that contained fewer than five taxa. In total, we use 448 758 gene trees in the PhyloNet analyses. We root the gene trees on the known outgroup (African hunting dog) or on dhole if African hunting dog is not in the gene tree.

## 3. Results

### 3.1 Simulation study on eight or fewer taxa

#### 3.1.1 Identifying the correct network as the top 1 ranked network with the smallest invariant score

**Figure 3.**
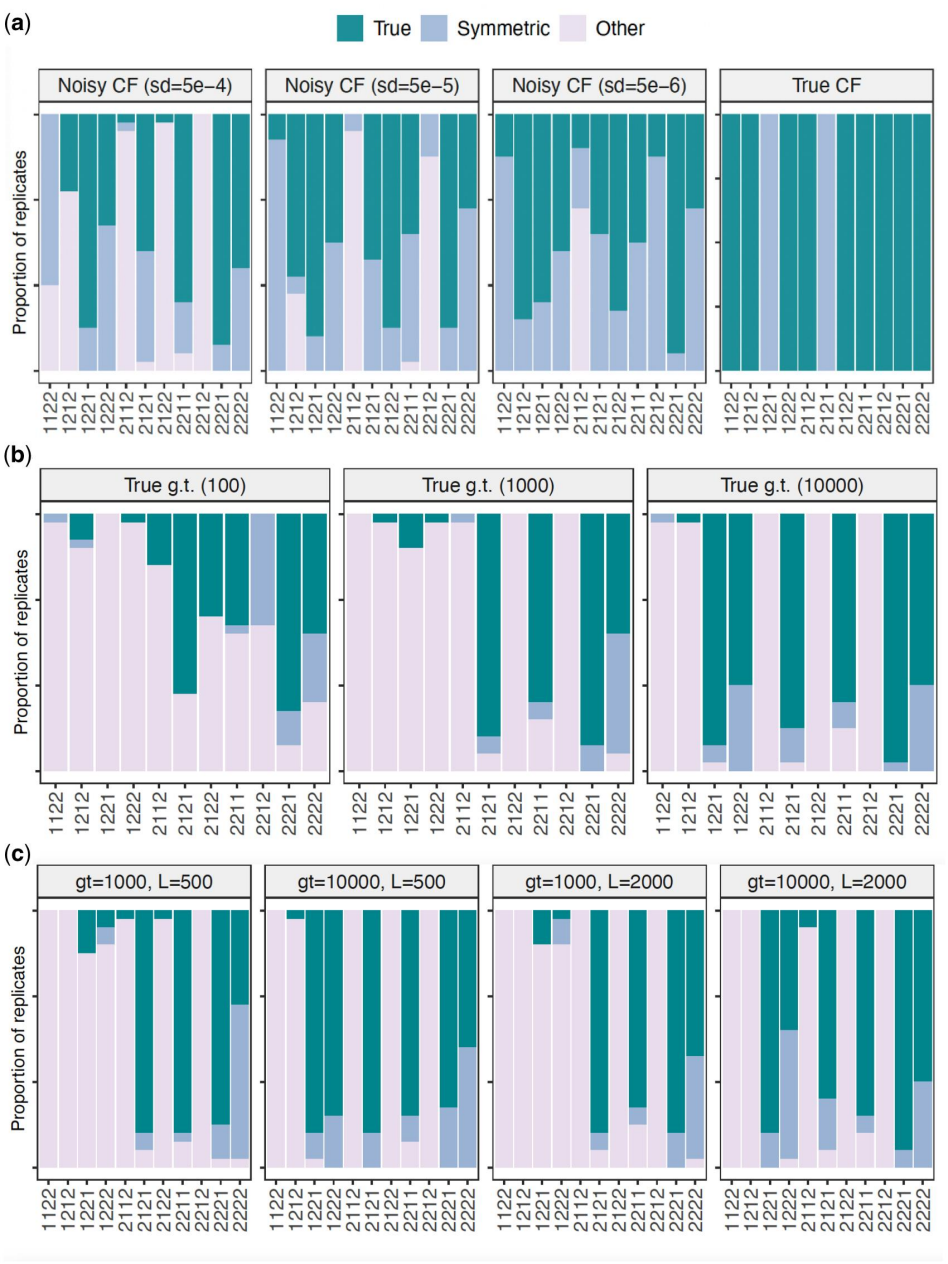
Proportion of times that the true hybridization cycle or the symmetric cycle (with clades $n_{1}$ and $n_{2}$ inverted) are in the top 1 ranked hybridization cycles identified by the phylogenetic invariants on three different cases. *Y*-axis is between 0 and 100. (a) True and Gaussian-perturbed CFs. Each panel corresponds to a type of simulation: using true concordance factors (right) and using concordance factors with added Gaussian noise (with decreasing standard deviation for noise from left to right). Whenever there are at least two taxa in each of the four clades (network 2222), our method is very accurate in detecting the true (or its symmetric) network. Our method is also accurate to identify the true network in the top 5 ranked networks (see Fig. 4a) which will greatly reduce the space of candidate networks to be compared with a likelihood approach. (b) true simulated gene trees (“g.t.”). Whenever there are at least two taxa in each of the four clades (network 2222), our method is very accurate in detecting the true (or its symmetric) network. Our method is also accurate to identify the true network in the top 5 ranked networks (see Fig. 4b) which will greatly reduce the space of candidate networks to be compared with a likelihood approach. (c) estimated gene trees. Each panel corresponds to a number of gene trees (g.t. from 1000 to 10 000) and sequence length (*L* from 500 bp to 2000 bp). Whenever there are at least two taxa in each of the four clades (network 2222) or only clade $n_{3}$ having one taxon (N=2221), our method is very accurate in detecting the true (or its symmetric) network. Our method is also accurate to identify the true network in the top 5 ranked networks (see Fig. 4c) which will greatly reduce the space of candidate networks to be compared with a likelihood approach.

Figure 3a shows the proportion of times (out of the 30 replicates) that our invariants method identifies the true network as the top 1 ranked network with the smallest invariant score for the cases of true concordance factors and the Gaussian-perturbed CFs with increasing standard deviation (from left to right). We also quantify the proportion of times that our invariants method identifies the symmetric network (when clades $n_{1}$ and $n_{2}$ in Fig. 1a are switched) because in the four-node hybridization cycle it is difficult for the method to distinguish clades $n_{1}$ and $n_{2}$ given that they both contribute to the hybridization node. Even for the case of true CFs, our method identifies the symmetric network as the top 1 network, instead of the true network, in two cases (N=1221 and N=2121), yet the difference in invariance score ($L_{2}$-norm of evaluated polynomials) of the true and symmetric networks in these cases is of the order of $10^{-16}$. For the noisiest setting (σ=0.0005, far left), it is evident that some networks are difficult to be detected by our method, but it is worth highlighting that whenever there are two taxa sampled on each of the four clades ($n_{0},n_{1},n_{2},n_{3}$) as in N=2222, our method always identifies the true (or symmetric) network as the top 1. This condition can easily be satisfied whenever there are multiple individuals per species.

Figure 3b shows the proportion of times (out of the 30 replicates) that our invariants method identifies the true network as the top 1 ranked network with smallest invariant score for the case of true simulated gene trees with increasing number of gene trees (from left to right), where “g.t.” is abbreviation for “gene trees”. Unlike the case of true or Gaussian-perturbed CFs, although our method in this case cannot identify all types of network structures with perfect accuracy, it can still identify the true (or symmetric) network when there are two taxa sampled per clade (N=2222) or at least two taxa sampled on the hybrid clade and sister clades (N=2221). With 1000 gene trees or more, the networks N=2121 and N=2211 are also accurately identified by our method.

Figure 3c shows the proportion of times (out of the 30 replicates) that our invariants method identifies the true network as the top 1 ranked network with smallest invariant score for the case of estimated gene trees with increasing number of gene trees and sequence length (from left to right). Again, although our method in this case cannot identify all types of network structures with perfect accuracy, it can still identify the true (or symmetric) network when there are two taxa sampled per clade (N=2222) or at least two taxa sampled on the hybrid clade and sister clades (N=2221). As before, with 1000 gene trees, the networks N=2121 and N=2211 are also accurately identified by our method, and with 10 000 gene trees, the networks N=1221 and N=1222 are also accurately identified.

Summarizing Fig. 3 related to our method’s ability to identify the true (or symmetric) network as top 1 ranked network by its invariant score, we can highlight that networks with only one sampled taxon in some of the clades are harder to be identified. As long as two taxa are sampled from each of the clades, our method is able to identify the network with high accuracy on all simulation settings. The number of genes is more important than the sequence length, and with at least 100 genes, our method is able to correctly identify networks with fewer than two taxa sampled from some clades (like 2221). We highlight that we are equally interested in the true and symmetric networks since a follow-up optimization of branch lengths and inheritance probability (γ) on the fixed network will estimate the correct γ and identify which of the sister clades is the major (γ>0.5) and which is the minor (γ<0.5).

#### 3.1.2 Reducing the space of candidate networks using the invariant score

**Figure 4.**
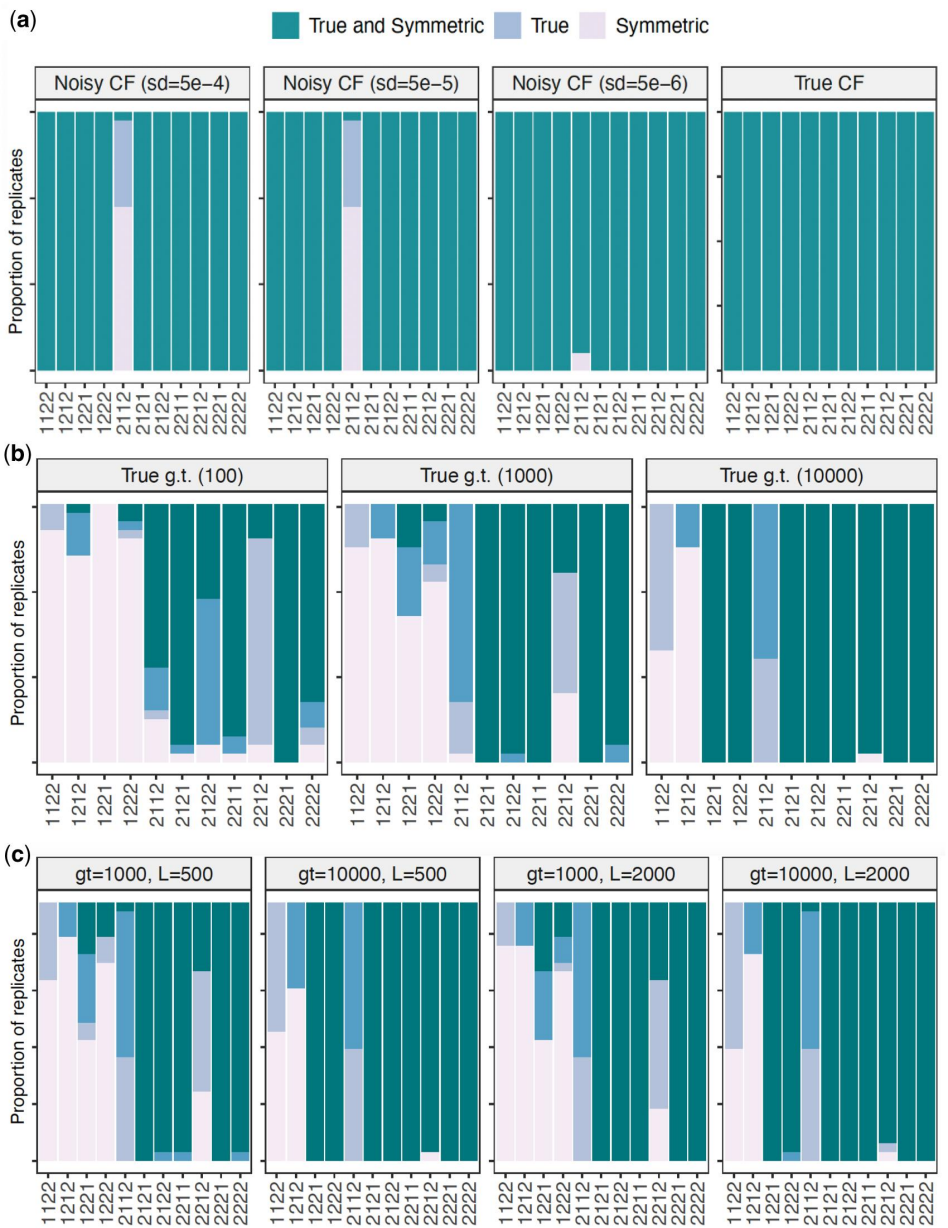
Proportion of times that the true network or the symmetric network (with clades $n_{1}$ and $n_{2}$ inverted) are in the top 5 ranked networks identified by the phylogenetic invariants on 3 different cases. *Y*-axis is between 0 and 100. (a) True and Gaussian-perturbed CFs. Each panel corresponds to a type of simulation: using true concordance factors (left) and using concordance factors with added Gaussian noise (with increasing standard deviation for noise from left to right). All networks are accurately identified in the top 5 ranked based on invariant score which provides evidence that our method fast and accurately reduce the space of candidate networks to just five alternatives. (b) True simulated gene trees (“g.t.”). Only the networks that have one taxon below the hybrid node (1122, 1212, 1221, 1222) do not allow accurate reconstruction with fewer than 10 000 gene trees which brings into attention the importance of taxon sampling for this method. **(c)** estimated gene trees. Each panel corresponds to a number of gene trees (g.t. from 1000 to 10 000) and sequence length (*L* from 500 to 2000 bp). Only the networks that have one taxon below the hybrid node (1122, 1212, 1221, 1222) do not allow accurate reconstruction with fewer than 10 000 gene trees which brings into attention the importance of taxon sampling for this method.

Figure 4a shows the proportion of times (out of the 30 replicates) that the true (or symmetric) network are within the top five ranked networks based on smallest invariant score for the cases of true concordance factors and the Gaussian-perturbed CFs with increasing standard deviation (from left to right). In all cases, our method includes the true (and symmetric) networks within the top five which means that our method accurately and in a fast manner reduces the space of candidate networks to just five candidates that can later be tested with another accurate methodology like likelihood.

Figure 4b shows the proportion of times (out of the 30 replicates) that the true (or symmetric) network are within the top five ranked networks based on smallest invariant score for the case of true simulated gene trees. Only the networks with one sampled taxon on the hybrid clade ($n_{0}$) cannot be recovered with 100 or 1000 gene trees. Whenever there are 10 000 gene trees in the sample, the networks N=1221 and N=1222 can now be recovered as part of the top 5, yet the networks N=1122 or N=1212 (when there is one sampled taxon in the hybrid clade and one of the sister clades) failed to be detected. The same conclusions are true for the case of estimated gene trees (Fig. 4c) with number of gene trees having more weight on the accuracy than sequence length.

Summarizing Fig. 4 related to our method’s ability to identify the true (or symmetric) network within the top five ranked networks by its invariant score, we can highlight that as long as there are at least two taxa sampled from the hybrid clade, then our method is able to place the true (and symmetric) network within the top five networks. This effectively reduces the space of candidate networks to compare with a likelihood approach. As mentioned, we do not view our method as a substitute to existing network inference methods. On the contrary, we believe that our invariants method will serve as a complement to identify a small subset of network possibilities that will help bypass the optimization on network space.

Supplementary Figure shows the rank in the invariant scores of the true and symmetric networks on the cases of true and Gaussian-perturbed CFs. Ideally, the true network (or at least its symmetric version) should be ranked as 1 by our invariants method. While the true and symmetric networks are not always ranked in number 1, they are ranked within the top 5 for most of the cases. This further confirms the advantage of our current method. It is a fast way to reduce the space of candidate networks (top 5) that can later be tested using a likelihood approach. The true (and symmetric) networks are not within the top 5 networks only for the cases of one sampled taxon from the hybrid clade (N=1122,1212,1221,1222). This same behavior is evident in for estimated gene trees. Again, these figures show our method’s ability to reduce the possible networks that fit the data.

In Supplementary Material, we also show plots with the values of the invariant scores for the true and symmetric networks.

Last, we present a comparison of the running times of four network methods in the Appendix. While all methods are able to identify the correct network, our phylogenetic invariants method (top row) only takes 7.17 s to correctly infer the network N=2222 which is over twice as fast as the second fastest (PhyloNet MPL (Yu and Nakhleh 2015)). The difference in running times (and even accuracy) is more evident in the results on the *Canis* dataset (Section 3.3).

### 3.2 Simulation study on more than eight taxa

Table in the Supplementary Material shows the proof-of-concept results on simulated data with more than eight taxa. Since the algorithm for more than eight taxa (Algorithm in the Supplementary Material) relies entirely on the algorithm for eight or fewer taxa (Algorithm 1), we simply show here that our steps to append taxon to the optimal subset of eight taxa identified by the method indeed yields the desired network with more than eight taxa.

### 3.3 Phylogenetic network for the Canis genus

**Figure 5.**
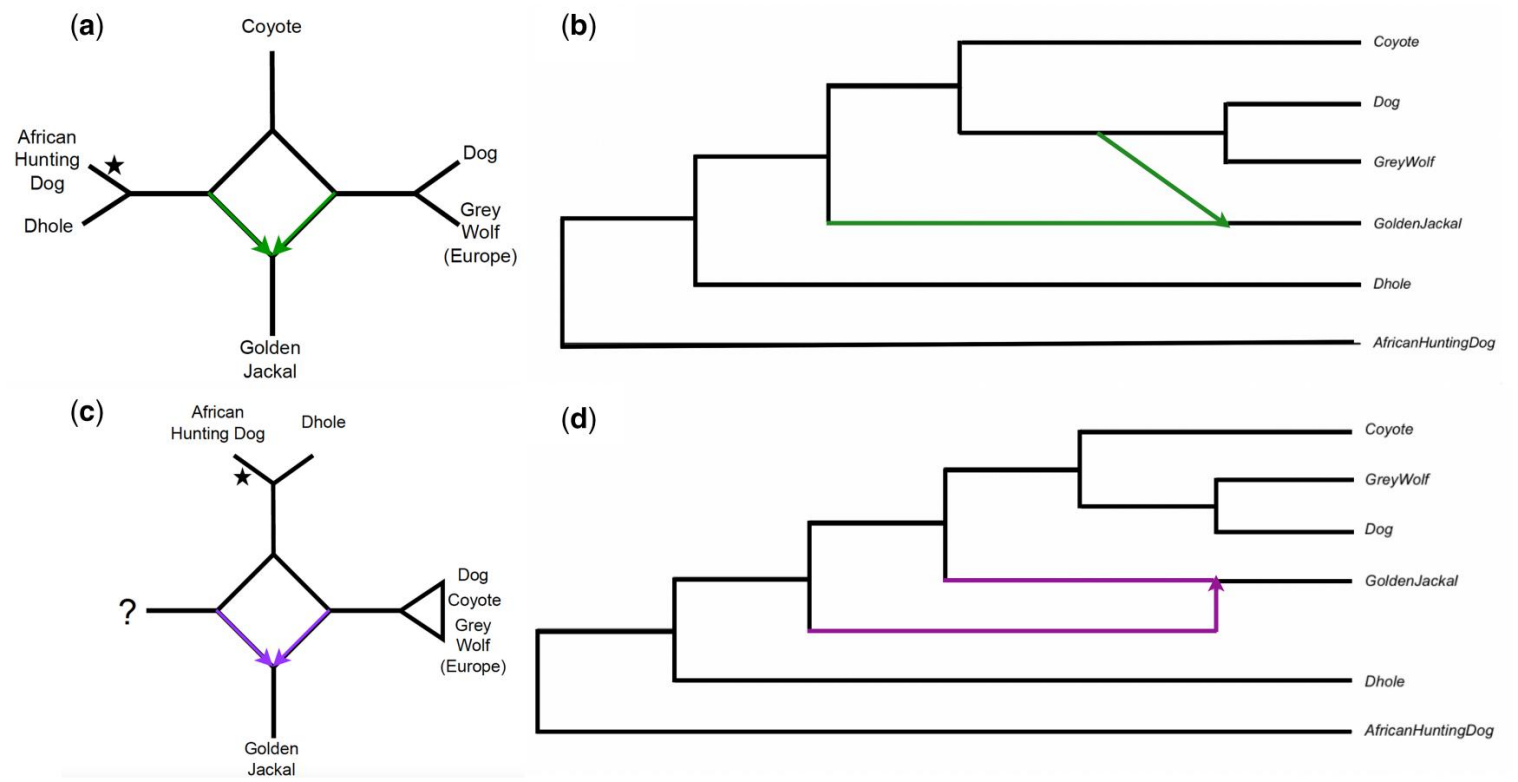
(a) Semi-directed phylogenetic network estimated by SNaQ (Solís-Lemus and Ané 2016), PhyloNet ML (Yu *et al.* 2014), and our method based on phylogenetic invariants. (b) Rooted version of the semi-directed networks estimated by SNaQ, PhyloNet ML, and our method based on phylogenetic invariants. (c) Semi-directed phylogenetic network estimated by PhyloNet MPL (Yu and Nakhleh 2015). The star marks the place where the root should be. (d) Rooted version of the semi-directed networks estimated by PhyloNet MPL. The network estimated with PhyloNet MPL (d) is different from the network inferred by the other methods, and it involves an unsampled (or extinct) taxon in the hybridization. SNaQ, PhyloNet ML, and our phylogenetic invariants method (a, b) identify the same hybridization event as the original publication (Gopalakrishnan *et al.* 2018) between the ancestral species to dog and grey wolf and the ancestral species of golden jackal, yet PhyloNet ML takes 389 times longer and SNaQ takes 20 times longer than our invariants method (see running times in Table 1).

The original analysis of the *Canis* genus identified nine gene flow events using the D statistics (Patterson *et al.* 2012) (Fig. 3a in Gopalakrishnan *et al.* (2018)). Here, we are able to replicate the hybridization event involving gene flow from the ancestor of dog and grey wolf into the ancestor of golden jackal (Fig. 5a and b). The same network is correctly inferred by SNaQ and by PhyloNet ML, but both methods take much longer to run (Table 1). PhyloNet MPL identifies a different hybridization event that has not been reported in previous studies, so it is impossible to validate it at this point. This hybridization involves an unsampled or extinct taxon (Fig. 5c and d).

**Table 1.** Running times (in seconds) on the *Canis* dataset.^a^

Method	Time (s)
Phylogenetic invariants (our method)	6.78
SNaQ	140.58
PhyloNet ML	2723.99
PhyloNet MPL	281.25

a Our method based on evaluation of phylogenetic invariants is 20 times faster than the second fastest method (SNaQ).

In terms of running time, our invariants method is able to identify the correct hybridization in under 7 s, while SNaQ (which also identifies the correct hybridization) takes over 140 s. PhyloNet MPL takes 40 times longer than our invariants method (with running time of over 281 s) and identifies a different hybridization event to those originally published (so, not possible to validate at this point). PhyloNet ML is the slowest method with over 45 min of running time on this small network of only six taxa. The inferred network by PhyloNet ML also agrees with the one originally published in Gopalakrishnan *et al.* (2018).

## 4. Discussion

Here, we introduce a novel method to infer four-node hybridization cycles on level-1 phylogenetic networks of over five taxa based on ultrafast evaluations of phylogenetic invariants. Our method bypasses optimization on network space and is able to accurately detect the hybridization cycles based on simulated and real data, especially when there are at least two taxa sampled from each of the four clades defined by the hybridization cycle (Fig. 1a). While our method is at least ∼10 times faster than other existing network methods, it is not meant to replace existing methods. On the contrary, we believe that our ultrafast algorithm can work in conjunction with existing methodologies by reducing the space of candidate networks that can later be evaluated based on likelihood or Bayesian approaches.

Nevertheless, there are limitations in our current method. Specifically, we are only able to detect hybridization cycles with four nodes on level-1 networks. For the case of three nodes in the hybridization cycle, it has been investigated already in Solís-Lemus and Ané (2016) that the set of phylogenetic invariants is empty. However, there is still room to extend the current methodology to hybridization cycles of five nodes or more. In addition, even when our method can infer networks with any number of taxa, it is unable to resolve the topology inside any of the four clades defined by the hybridization cycle (Fig. 1a). That is, our method is only able to identify the placement of the four-node hybridization cycle. For this reason, we view our method as in line with other hybrid detection methods such as ABBA-BABA test (Patterson *et al.* 2012), MSCquartets (Mitchell *et al.* 2019, Rhodes *et al.* 2021), and HyDe (Kubatko and Chifman 2019) that are also only able to detect specific hybridization patterns in subsets of taxa.

Future work will involve the generation of the phylogenetic invariants related to hybridization cycles of five nodes or more, and the development of a merging algorithm that can produced a fully resolved *n*-taxon phylogenetic network from the eight-taxon estimated networks inferred by our invariants method. In addition, our proposed method is unable to infer biological parameters like inheritance probability or branch length, but future work could exploit the CF formulas (which depend on branch lengths and inheritance probabilities) to provide estimated values to these parameters from the observed CFs.

## Supplementary Material

vbae014_Supplementary_Data

## Data Availability

The *Canis* dataset was made publicly available by the original publication (Gopalakrishnan *et al.* 2018) and can be accessed through the GitHub repository of Zhang and Mirarab (2022) here https://github.com/chaoszhang/Weighted-ASTRAL_data. All the scripts for our work are publicly available in the GitHub repository https://github.com/solislemuslab/PhyloDiamond.jl. The new Julia package PhyloDiamond.jl is open-source, publicly available in https://github.com/solislemuslab/PhyloDiamond.jl.

## References

[vbae014-B1] Adavoudi R , Pilot M. Consequences of hybridization in mammals: a systematic review. Genes (Basel) 2021;13:50. 35052393 10.3390/genes13010050 PMC8774782

[vbae014-B2] Allman ES , Rhodes JA. Phylogenetic invariants for the general Markov model of sequence mutation. Math Biosci 2003;186:113–44. 14583169 10.1016/j.mbs.2003.08.004

[vbae014-B3] Ardiyansyah M. Distinguishing level-2 phylogenetic networks using phylogenetic invariants. arXiv, arXiv:2104.12479, 2021, preprint: not peer reviewed.

[vbae014-B4] Barton T, Gross E, Long C, Rusinko J. Statistical learning with phylogenetic network invariants. arXiv, arXiv:2211.11919, 2022, preprint: not peer reviewed.

[vbae014-B5] Baum DA. Concordance trees, concordance factors, and the exploration of reticulate genealogy. Taxon 2007;56:417–26. http://www.jstor.org/stable/25065797.

[vbae014-B6] Bjorner M , Molloy EK, Dewey CN, Solis-Lemus C. Detectability of varied hybridization scenarios using genome-scale hybrid detection methods. arXiv, arXiv:2211.00712, 2022, preprint: not peer reviewed.

[vbae014-B7] Blair C , Ané C. Phylogenetic trees and networks can serve as powerful and complementary approaches for analysis of genomic data. Syst Biol 2020;69:593–601. 31432090 10.1093/sysbio/syz056

[vbae014-B8] Bryant D , Moulton V. Neighbor-Net: an agglomerative method for the construction of phylogenetic networks. Mol Biol Evol 2004;21:255–65. 14660700 10.1093/molbev/msh018

[vbae014-B9] Casanellas M , Fernández-Sánchez J, Garrote-López M et al Designing weights for Quartet-Based methods when data are heterogeneous across lineages. Bull Math Biol 2023;85:68. 37310552 10.1007/s11538-023-01167-y PMC10264505

[vbae014-B10] Casanellas M , Fernandez-Sanchez J, Garrote-Lopez M et al SAQ: semi-Algebraic quartet reconstruction. IEEE/ACM Trans Comput Biol Bioinform 2021;18:2855–61. 34339375 10.1109/TCBB.2021.3101278

[vbae014-B11] Charles KM , Stehlik I. Assisted species migration and hybridization to conserve cold-adapted plants under climate change. Conserv Biol 2021;35:559–66. 32643822 10.1111/cobi.13583

[vbae014-B12] Cummings J, Hollering B, Manon C. Invariants for level-1 phylogenetic networks under the cavendar-farris-neyman model. *Adv Appl Math* 2024;153:102633.

[vbae014-B13] De Santis V , Quadroni S, Britton RJ et al Biological and trophic consequences of genetic introgression between endemic and invasive Barbus fishes. Biol Invasions 2021;23:3351–68. 34054333 10.1007/s10530-021-02577-6 PMC8149140

[vbae014-B14] Degnan JH. Modeling hybridization under the network multispecies coalescent. Syst Biol 05 2018;67:786–99. 10.1093/sysbio/syy040. 29846734 PMC6101600

[vbae014-B15] Diop A , Torrance EL, Stott CM et al Gene flow and introgression are pervasive forces shaping the evolution of bacterial species. Genome Biol 2022;23:239. 36357919 10.1186/s13059-022-02809-5 PMC9650840

[vbae014-B16] Elworth RAL, Ogilvie HW, Zhu J, Nakhleh L. Advances in computational methods for phylogenetic networks in the presence of hybridization. In: Warnow T (ed.) Bioinformatics and Phylogenetics. Springer, 2019, 317–360.

[vbae014-B17] Felsenstein J. Counting phylogenetic invariants in some simple cases. J Theor Biol 1991;152:357–76. 1749255 10.1016/s0022-5193(05)80200-0

[vbae014-B18] Fernández-Sánchez J , Casanellas M. Invariant versus classical quartet inference when evolution is heterogeneous across sites and lineages. Syst Biol 2016;65:280–91. 26559009 10.1093/sysbio/syv086

[vbae014-B19] Gopalakrishnan S , Sinding M-HS, Ramos-Madrigal J et al Interspecific gene flow shaped the evolution of the genus canis. Current Biology 2018;28:3441–9.e5. 30344120 10.1016/j.cub.2018.08.041 PMC6224481

[vbae014-B20] Grayson D , Stillman M. Macaulay2, a software system for research in algebraic geometry. http://www.math.uiuc.edu/Macaulay2/ (January 2022, date last accessed).

[vbae014-B21] Gross E , Long C. Distinguishing phylogenetic networks. SIAM J Appl Algebra Geometry 2018;2:72–93. 10.1137/17M1134238.

[vbae014-B22] Gross E , van Iersel L, Janssen R et al Distinguishing level-1 phylogenetic networks on the basis of data generated by Markov processes. J Math Biol 2021;83:32. 34482446 10.1007/s00285-021-01653-8 PMC8418599

[vbae014-B23] Grünewald S, Forslund K, Dress A, Moulton V. QNet: an agglomerative method for the construction of phylogenetic networks from weighted quartets. Mol Biol Evol 2007;24:532–8. 17119010 10.1093/molbev/msl180

[vbae014-B24] Hudson RR. Generating samples under a wright–fisher neutral model of genetic variation. Bioinformatics 2002;18:337–8. 11847089 10.1093/bioinformatics/18.2.337

[vbae014-B25] Huson D, Rupp R, Scornavacca C. Phylogenetic Networks, 1st edn. New York, NY: Cambridge University Press, 2010.

[vbae014-B26] Huson DH , Bryant D. Application of phylogenetic networks in evolutionary studies. Mol Biol Evol 2006;23:254–67. 16221896 10.1093/molbev/msj030

[vbae014-B27] Kalyaanamoorthy S , Quang Minh B, Wong TKF et al modelfinder: fast model selection for accurate phylogenetic estimates. Nat Methods 2017;14:587–9. 28481363 10.1038/nmeth.4285 PMC5453245

[vbae014-B28] Kong S , Pons JC, Kubatko L et al Classes of explicit phylogenetic networks and their biological and mathematical significance. J Math Biol 2022;84:47–4. 35503141 10.1007/s00285-022-01746-y

[vbae014-B29] Kubatko LS , Chifman J. An invariants-based method for efficient identification of hybrid species from large-scale genomic data. BMC Evol Biol 2019;19:112. 10.1186/s12862-019-1439-7. 31146685 PMC6543680

[vbae014-B30] Minh BQ , Schmidt HA, Chernomor O et al iq-tree 2: new models and efficient methods for phylogenetic inference in the genomic era. Mol Biol Evol, 2020;37:1530–4. 32011700 10.1093/molbev/msaa015 PMC7182206

[vbae014-B31] Mitchell JD , Allman ES, Rhodes JA. Hypothesis testing near singularities and boundaries. Electron J Stat 2019;13:2150–93. 10.1214/19-ejs1576 33163140 PMC7643865

[vbae014-B32] Pang X-X , Zhang D-Y. Impact of ghost introgression on coalescent-based species tree inference and estimation of divergence time. *Syst Biol* 2023;72:35–49. 10.1093/sysbio/syac047 35799362

[vbae014-B33] Patterson N , Moorjani P, Luo Y et al Ancient admixture in human history. Genetics 2012;192:1065–93. 10.1534/genetics.112.145037 22960212 PMC3522152

[vbae014-B34] Pérez-Escobar OA , Bellot S, Przelomska NAS et al Molecular clocks and archeogenomics of a late period Egyptian date palm leaf reveal introgression from wild relatives and add timestamps on the domestication. Mol Biol Evol 2021;38:4475–92. 34191029 10.1093/molbev/msab188 PMC8476131

[vbae014-B35] Rambaut A , Grass NC. Seq-Gen: an application for the Monte Carlo simulation of DNA sequence evolution along phylogenetic trees. Comput Appl Biosci 1997;13:235–8. 9183526 10.1093/bioinformatics/13.3.235

[vbae014-B36] Rey O , Toulza E, Chaparro C et al Diverging patterns of introgression from *Schistosoma bovis* across *S. haematobium* African lineages. PLoS Pathog 2021;17:e1009313. 33544762 10.1371/journal.ppat.1009313 PMC7891765

[vbae014-B37] Rhodes JA , Baños H, Mitchell JD et al MSCquartets 1.0: quartet methods for species trees and networks under the multispecies coalescent model in R. Bioinformatics 2021;37:1766–8. 10.1093/bioinformatics/btaa868 33031510 PMC8289379

[vbae014-B38] Solís-Lemus C , Ané C. Inferring phylogenetic networks with maximum pseudolikelihood under incomplete lineage sorting. PLoS Genet 2016;12:e1005896. 26950302 10.1371/journal.pgen.1005896 PMC4780787

[vbae014-B39] Solís-Lemus C , Bastide P, Ané C et al PhyloNetworks: a package for phylogenetic networks. Mol Biol Evol 2017;34:3292–8. 28961984 10.1093/molbev/msx235

[vbae014-B40] Solís-Lemus C , Yang M, Ané C et al Inconsistency of species tree methods under gene flow. Syst Biol 2016;65:843–51. Sept. 27151419 10.1093/sysbio/syw030

[vbae014-B41] Solís-Lemus CR , Coen A, Ané C. On the Identifiability of Phylogenetic Networks under a Pseudolikelihood model. arXiv, arXiv:2010.01758, 2020, preprint: not peer reviewed.

[vbae014-B42] Steel MA , Fu Y. Classifying and counting linear phylogenetic invariants for the Jukes–Cantor model. J Comput Biol 1995;2:39–47. 7497119 10.1089/cmb.1995.2.39

[vbae014-B43] Steensels J , Gallone B, Verstrepen KJ et al Interspecific hybridization as a driver of fungal evolution and adaptation. Nat Rev Microbiol 2021;19:485–500. 33767366 10.1038/s41579-021-00537-4

[vbae014-B44] Suvorov A , Scornavacca C, Fujimoto MS et al Deep ancestral introgression shapes evolutionary history of dragonflies and damselflies. Syst Biol 2022;71:526–46. 34324671 10.1093/sysbio/syab063 PMC9017697

[vbae014-B45] Tricou T, Tannier E, de Vienne DM. Ghost lineages highly influence the interpretation of introgression tests. *Syst Biol* 2022;71:1147–58. 10.1093/sysbio/syac011 PMC9366450 35169846

[vbae014-B46] Wen D , Yu Y, Nakhleh L et al Bayesian inference of reticulate phylogenies under the multispecies network coalescent. PLoS Genet 2016;12:e1006006. 27144273 10.1371/journal.pgen.1006006 PMC4856265

[vbae014-B47] Yu Y , Dong J, Liu KJ et al Maximum likelihood inference of reticulate evolutionary histories. Proc Natl Acad Sci USA 2014;111:16448–53. 10.1073/pnas.1407950111 25368173 PMC4246314

[vbae014-B48] Yu Y , Nakhleh L. A maximum pseudo-likelihood approach for phylogenetic networks. BMC Genomics 2015;16 Suppl 10:S10. 10.1186/1471-2164-16-S10-S10 PMC4602316 26450642

[vbae014-B49] Zhang C , Mirarab S. Weighting by gene tree uncertainty improves accuracy of quartet-based species trees. Mol Biol Evol 2022;39:msac215. 36201617 10.1093/molbev/msac215 PMC9750496

[vbae014-B50] Zhang C , Rabiee M, Sayyari E et al ASTRAL-III: polynomial time species tree reconstruction from partially resolved gene trees. BMC Bioinformatics 2018;19:153. 29745866 10.1186/s12859-018-2129-y PMC5998893

