# Supplementary material for: Ultrafast learning of four-node hybridization cycles in phylogenetic networks using algebraic invariants
Source: Bioinform Adv. 2024 Feb 8;4(1):vbae014. doi: 10.1093/bioadv/vbae014 (PMC10879748; doi:10.1093/bioadv/vbae014)
Supplement: vbae014_Supplementary_Data [file vbae014_supplementary_data.pdf]

# SUPPLEMENTAL MATERIAL

## Representation of a level-1 semi-directed phylogenetic network with a set of polynomial equations corresponding to the expected concordance factors under the coalescent model

Notation:

- $n$ corresponds to the number of individuals from each of the 4 clades: $(n_0, n_1, n_2, n_3)$. For example, $n = (0, 0, 2, 2)$ means that you have the quartet with 2 individuals in $n_2$ and 2 individuals in $n_3$
- "Individuals" list the individuals taken from each of the four clades. For example, $i_1, i_2 \in n_0$ means that we took two species from clade $n_0$ denoted $i_1$ and $i_2$. We use these species names to define the splits for the formulas: e.g. $P(i_1, i_2|j_1, j_2)$ represents the probability that $i_1$ and $i_2$ are together in one side of the split (and $j_1, j_2$ together)
- "Type" corresponds to the type of quartet which matches the types in [1]
- "CF Formula" corresponds to the formula of the expected CF for that given quartet under the multispecies coalescent model
- "CF value" is the variable we give to the observed CF we will read from the data table

By [2], we know that we only need to select at most two individuals per subgraph $(n_0, n_1, n_2, n_3)$ to define all the CF formulas that involve the hybridization cycle. We list all the CF formulas in the table below assuming that we do have 2 individuals per clade $(n_0, n_1, n_2, n_3)$ (that is, we have at least eight species).

| $n$ | Type | CF Formula | CF value |
|---|---|---|---|
| $(0, 0, 2, 2)$ | 5 | $1 - \frac{2}{3} z_2 z_{2,3} z_3$ | $a1$ |
| | | $\frac{1}{3} z_2 z_{2,3} z_3$ | $a2$ |
| | | $\frac{1}{3} z_2 z_{2,3} z_3$ | $a3$ |
| $(0, 1, 2, 1)$ | 5 | $1 - \frac{2}{3} z_{2,3} z_2$ | $a4$ |
| | | $\frac{1}{3} z_{2,3} z_2$ | $a5$ |
| | | $\frac{1}{3} z_{2,3} z_2$ | $a6$ |
| $(0, 1, 1, 2)$ | 5 | $1 - \frac{2}{3} z_3$ | $a7$ |
| | | $\frac{1}{3} z_3$ | $a8$ |
| | | $\frac{1}{3} z_3$ | $a9$ |
| $(0, 2, 2, 0)$ | 5 | $1 - \frac{2}{3} z_2 z_{2,3} z_{1,3} z_1$ | $a10$ |
| | | $\frac{1}{3} z_2 z_{2,3} z_{1,3} z_1$ | $a11$ |
| | | $\frac{1}{3} z_2 z_{2,3} z_{1,3} z_1$ | $a12$ |
| $(0, 2, 1, 1)$ | 5 | $1 - \frac{2}{3} z_{1,3} z_1$ | $a13$ |
| | | $\frac{1}{3} z_{1,3} z_1$ | $a14$ |
| | | $\frac{1}{3} z_{1,3} z_1$ | $a15$ |
| $(0, 2, 0, 2)$ | 5 | $1 - \frac{2}{3} z_3 z_{1,3} z_1$ | $a16$ |
| | | $\frac{1}{3} z_3 z_{1,3} z_1$ | $a17$ |
| | | $\frac{1}{3} z_3 z_{1,3} z_1$ | $a18$ |
| $(1, 0, 2, 1)$ | 2 | $(1-\gamma)\left(1 - \frac{2}{3} z_{2,3} z_2\right) + \gamma\left(1 - \frac{2}{3} z_2\right)$ | $a19$ |
| | | $(1-\gamma)\frac{1}{3} z_{2,3} z_2 + \gamma \frac{1}{3} z_2$ | $a20$ |
| | | $(1-\gamma)\frac{1}{3} z_{2,3} z_2 + \gamma \frac{1}{3} z_2$ | $a21$ |
| $(1, 0, 1, 2)$ | 2 | $(1-\gamma)\left(1 - \frac{2}{3} z_3\right) + \gamma\left(1 - \frac{2}{3} z_{2,3} z_3\right)$ | $a22$ |
| | | $(1-\gamma)\frac{1}{3} z_3 + \gamma \frac{1}{3} z_{2,3} z_3$ | $a23$ |
| | | $(1-\gamma)\frac{1}{3} z_3 + \gamma \frac{1}{3} z_{2,3} z_3$ | $a24$ |
| $(1, 1, 2, 0)$ | 2 | $(1-\gamma)\left(1 - \frac{2}{3} z_{1,3} z_{2,3} z_2\right) + \gamma\left(1 - \frac{2}{3} z_2\right)$ | $a25$ |
| | | $(1-\gamma)\frac{1}{3} z_{1,3} z_{2,3} z_2 + \gamma \frac{1}{3} z_2$ | $a26$ |
| | | $(1-\gamma)\frac{1}{3} z_{1,3} z_{2,3} z_2 + \gamma \frac{1}{3} z_2$ | $a27$ |

| | | | |
|---|---|---|---|
| (1, 1, 1, 1) | 3 | $(1-\gamma)\left(1-\frac{2}{3}z_{1,3}\right)+\gamma\frac{1}{3}z_{2,3}$ | $a28$ |
| | | $(1-\gamma)\frac{1}{3}z_{1,3}+\gamma\left(1-\frac{2}{3}z_{2,3}\right)$ | $a29$ |
| | | $(1-\gamma)\frac{1}{3}z_{1,3}+\gamma\frac{1}{3}z_{2,3}$ | $a30$ |
| (1, 1, 0, 2) | 2 | $(1-\gamma)\left(1-\frac{2}{3}z_{1,3}z_3\right)+\gamma\left(1-\frac{2}{3}z_3\right)$ | $a31$ |
| | | $(1-\gamma)\frac{1}{3}z_{1,3}z_3+\gamma\frac{1}{3}z_3$ | $a32$ |
| | | $(1-\gamma)\frac{1}{3}z_{1,3}z_3+\gamma\frac{1}{3}z_3$ | $a33$ |
| (1, 2, 1, 0) | 2 | $(1-\gamma)\left(1-\frac{2}{3}z_1\right)+\gamma\left(1-\frac{2}{3}z_{2,3}z_{1,3}z_1\right)$ | $a34$ |
| | | $(1-\gamma)\frac{1}{3}z_1+\gamma\frac{1}{3}z_{2,3}z_{1,3}z_1$ | $a35$ |
| | | $(1-\gamma)\frac{1}{3}z_1+\gamma\frac{1}{3}z_{2,3}z_{1,3}z_1$ | $a36$ |
| (1, 2, 0, 1) | 2 | $(1-\gamma)\left(1-\frac{2}{3}z_1\right)+\gamma\left(1-\frac{2}{3}z_{1,3}z_1\right)$ | $a37$ |
| | | $(1-\gamma)\frac{1}{3}z_1+\gamma\frac{1}{3}z_{1,3}z_1$ | $a38$ |
| | | $(1-\gamma)\frac{1}{3}z_1+\gamma\frac{1}{3}z_{1,3}z_1$ | $a39$ |
| (2, 0, 2, 0) | 4 | $(1-\gamma)^2\left(1-\frac{2}{3}z_2z_0z_{0,1}z_{1,3}z_{2,3}\right)+2\gamma(1-\gamma)\left(1-\frac{2}{3}z_2z_0\right)+\gamma^2\left(1-\frac{2}{3}z_2z_0z_{0,2}\right)$ | $a40$ |
| | | $(1-\gamma)^2\frac{1}{3}z_2z_0z_{0,1}z_{1,3}z_{2,3}+2\gamma(1-\gamma)\frac{1}{3}z_2z_0+\gamma^2\frac{1}{3}z_2z_0z_{0,2}$ | $a41$ |
| | | $(1-\gamma)^2\frac{1}{3}z_2z_0z_{0,1}z_{1,3}z_{2,3}+2\gamma(1-\gamma)\frac{1}{3}z_2z_0+\gamma^2\frac{1}{3}z_2z_0z_{0,2}$ | $a42$ |
| (2, 0, 1, 1) | 1 | $(1-\gamma)^2\left(1-\frac{2}{3}z_0z_{1,3}z_{0,1}\right)+2\gamma(1-\gamma)\left(1-z_0+\frac{1}{3}z_0z_{2,3}\right)+\gamma^2\left(1-\frac{2}{3}z_0z_{0,2}\right)$ | $a43$ |
| | | $(1-\gamma)^2\frac{1}{3}z_0z_{1,3}z_{0,1}+\gamma(1-\gamma)z_0\left(1-\frac{1}{3}z_{2,3}\right)+\gamma^2\frac{1}{3}z_0z_{0,2}$ | $a44$ |
| | | $(1-\gamma)^2\frac{1}{3}z_0z_{1,3}z_{0,1}+\gamma(1-\gamma)z_0\left(1-\frac{1}{3}z_{2,3}\right)+\gamma^2\frac{1}{3}z_0z_{0,2}$ | $a45$ |
| (2, 0, 0, 2) | 4 | $(1-\gamma)^2\left(1-\frac{2}{3}z_3z_0z_{1,3}z_{0,1}\right)+2\gamma(1-\gamma)\left(1-\frac{2}{3}z_3z_0\right)+\gamma^2\left(1-\frac{2}{3}z_3z_0z_{2,3}z_{0,2}\right)$ | $a46$ |
| | | $(1-\gamma)^2\frac{1}{3}z_3z_0z_{1,3}z_{0,1}+2\gamma(1-\gamma)\frac{1}{3}z_3z_0+\gamma^2\frac{1}{3}z_3z_0z_{2,3}z_{0,2}$ | $a47$ |
| | | $(1-\gamma)^2\frac{1}{3}z_3z_0z_{1,3}z_{0,1}+2\gamma(1-\gamma)\frac{1}{3}z_3z_0+\gamma^2\frac{1}{3}z_3z_0z_{2,3}z_{0,2}$ | $a48$ |
| (2, 1, 1, 0) | 1 | $(1-\gamma)^2\left(1-\frac{2}{3}z_0z_{0,1}\right)+2\gamma(1-\gamma)\left(1-z_0+\frac{1}{3}z_0z_{2,3}z_{1,3}\right)+\gamma^2\left(1-\frac{2}{3}z_0z_{0,2}\right)$ | $a49$ |
| | | $(1-\gamma)^2\frac{1}{3}z_0z_{0,1}+\gamma(1-\gamma)z_0\left(1-\frac{1}{3}z_{2,3}z_{1,3}\right)+\gamma^2\frac{1}{3}z_0z_{0,2}$ | $a50$ |
| | | $(1-\gamma)^2\frac{1}{3}z_0z_{0,1}+\gamma(1-\gamma)z_0\left(1-\frac{1}{3}z_{2,3}z_{1,3}\right)+\gamma^2\frac{1}{3}z_0z_{0,2}$ | $a51$ |
| (2, 1, 0, 1) | 1 | $(1-\gamma)^2\left(1-\frac{2}{3}z_0z_{0,1}\right)+2\gamma(1-\gamma)\left(1-z_0+\frac{1}{3}z_0z_{1,3}\right)+\gamma^2\left(1-\frac{2}{3}z_0z_{0,2}z_{2,3}\right)$ | $a52$ |
| | | $(1-\gamma)^2\frac{1}{3}z_0z_{0,1}+\gamma(1-\gamma)z_0\left(1-\frac{1}{3}z_{1,3}\right)+\gamma^2\frac{1}{3}z_0z_{0,2}z_{2,3}$ | $a53$ |
| | | $(1-\gamma)^2\frac{1}{3}z_0z_{0,1}+\gamma(1-\gamma)z_0\left(1-\frac{1}{3}z_{1,3}\right)+\gamma^2\frac{1}{3}z_0z_{0,2}z_{2,3}$ | $a54$ |
| (2, 2, 0, 0) | 4 | $(1-\gamma)^2\left(1-\frac{2}{3}z_1z_0z_{0,1}\right)+2\gamma(1-\gamma)\left(1-\frac{2}{3}z_1z_0\right)+\gamma^2\left(1-\frac{2}{3}z_1z_0z_{0,2}z_{2,3}z_{1,3}\right)$ | $a55$ |
| | | $(1-\gamma)^2\frac{1}{3}z_1z_0z_{0,1}+2\gamma(1-\gamma)\frac{1}{3}z_1z_0+\gamma^2\frac{1}{3}z_1z_0z_{0,2}z_{2,3}z_{1,3}$ | $a56$ |
| | | $(1-\gamma)^2\frac{1}{3}z_1z_0z_{0,1}+2\gamma(1-\gamma)\frac{1}{3}z_1z_0+\gamma^2\frac{1}{3}z_1z_0z_{0,2}z_{2,3}z_{1,3}$ | $a57$ |

Table 1: Concordance factor equations for all 4-taxon subsets that involve the hybridization cycle.

For the mapping of observed CFs to $a_i$ values, we need to know to which of the three splits each of the $a_i$'s corresponds to. So, next, we elaborate on which specific split each of the $a_i$ corresponds to:

$n=(0,0,2,2)$

- Individuals $k_1, k_2 \in n_2$ and $l_1, l_2 \in n_3$
- $P(k_1,k_2|l_1,l_2) = 1-\frac{2}{3}z_2z_{2,3}z_3 = a_1$
- $P(k_1,l_1|k_2,l_2) = \frac{1}{3}z_2z_{2,3}z_3 = a_2$
- $P(k_1,l_2|k_2,l_1) = \frac{1}{3}z_2z_{2,3}z_3 = a_3$

$n=(0,1,2,1)$

- Individuals $j_1 \in n_1$; $k_1, k_2 \in n_2$ and $l_1 \in n_3$
- $P(k_1,k_2|j_1,l_1) = 1-\frac{2}{3}z_2z_{2,3} = a_4$
- $P(k_1,j_1|k_2,l_1) = \frac{1}{3}z_2z_{2,3} = a_5$

- $P(k_1, l_1|k_2, j_1) = \frac{1}{3}z_2 z_{2,3} = a_6$

$n = (0, 1, 1, 2)$

- Individuals $j_1 \in n_1$; $k_1 \in n_2$ and $l_1, l_2 \in n_3$
- $P(l_1, l_2|j_1, k_1) = 1 - \frac{2}{3}z_3 = a_7$
- $P(l_1, j_1|l_2, k_1) = \frac{1}{3}z_3 = a_8$
- $P(l_1, k_1|l_2, j_1) = \frac{1}{3}z_3 = a_9$

$n = (0, 2, 2, 0)$

- Individuals $j_1, j_2 \in n_1$; $k_1, k_2 \in n_2$
- $P(j_1, j_2|k_1, k_2) = 1 - \frac{2}{3}z_2 z_{2,3} z_{1,3} z_1 = a_{10}$
- $P(j_1, k_1|j_2, k_2) = \frac{1}{3}z_2 z_{2,3} z_{1,3} z_1 = a_{11}$
- $P(j_1, k_2|j_2, k_1) = \frac{1}{3}z_2 z_{2,3} z_{1,3} z_1 = a_{12}$

$n = (0, 2, 1, 1)$

- Individuals $j_1, j_2 \in n_1$; $k_1 \in n_2$; $l_1 \in n_3$
- $P(j_1, j_2|k_1, l_1) = 1 - \frac{2}{3}z_{1,3} z_1 = a_{13}$
- $P(j_1, k_1|j_2, l_1) = \frac{1}{3}z_{1,3} z_1 = a_{14}$
- $P(j_1, l_1|j_2, k_1) = \frac{1}{3}z_{1,3} z_1 = a_{15}$

$n = (0, 2, 0, 2)$

- Individuals $j_1, j_2 \in n_1$; $l_1, l_2 \in n_3$
- $P(j_1, j_2|l_1, l_2) = 1 - \frac{2}{3}z_3 z_{1,3} z_1 = a_{16}$
- $P(j_1, l_1|j_2, l_2) = \frac{1}{3}z_3 z_{1,3} z_1 = a_{17}$
- $P(j_1, l_2|j_2, l_1) = \frac{1}{3}z_3 z_{1,3} z_1 = a_{18}$

$n = (1, 0, 2, 1)$

- Individuals $i_1 \in n_0$; $k_1, k_2 \in n_2$; $l_1 \in n_3$
- $P(k_1, k_2|i_1, l_1) = (1 - \gamma)\left(1 - \frac{2}{3}z_{2,3} z_2\right) + \gamma\left(1 - \frac{2}{3}z_2\right) = a_{19}$
- $P(k_1, i_1|k_2, l_1) = (1 - \gamma)\frac{1}{3}z_{2,3} z_2 + \gamma\frac{1}{3}z_2 = a_{20}$
- $P(k_1, l_1|k_2, i_1) = (1 - \gamma)\frac{1}{3}z_{2,3} z_2 + \gamma\frac{1}{3}z_2 = a_{21}$

$n = (1, 0, 1, 2)$

- Individuals $i_1 \in n_0$; $k_1 \in n_2$; $l_1, l_2 \in n_3$
- $P(l_1, l_2|i_1, k_1) = (1 - \gamma)\left(1 - \frac{2}{3}z_3\right) + \gamma\left(1 - \frac{2}{3}z_{2,3} z_3\right) = a_{22}$
- $P(l_1, i_1|l_2, k_1) = (1 - \gamma)\frac{1}{3}z_3 + \gamma\frac{1}{3}z_{2,3} z_3 = a_{23}$
- $P(l_1, k_1|l_2, i_1) = (1 - \gamma)\frac{1}{3}z_3 + \gamma\frac{1}{3}z_{2,3} z_3 = a_{24}$

$n = (1, 1, 2, 0)$

- Individuals $i_1 \in n_0$; $j_1 \in n_1$; $k_1, k_2 \in n_2$
- $P(k_1, k_2|i_1, j_1) = (1 - \gamma)\left(1 - \frac{2}{3}z_{1,3} z_{2,3} z_2\right) + \gamma\left(1 - \frac{2}{3}z_2\right) = a_{25}$
- $P(k_1, i_1|k_2, j_1) = (1 - \gamma)\frac{1}{3}z_{1,3} z_{2,3} z_2 + \gamma\frac{1}{3}z_2 = a_{26}$
- $P(k_1, j_1|k_2, i_1) = (1 - \gamma)\frac{1}{3}z_{1,3} z_{2,3} z_2 + \gamma\frac{1}{3}z_2 = a_{27}$

$n = (1, 1, 1, 1)$

- Individuals $i_1 \in n_0$; $j_1 \in n_1$; $k_1 \in n_2$; $l_1 \in n_3$
- $P(i_1, j_1|k_1, l_1) = (1 - \gamma)\left(1 - \frac{2}{3}z_{1,3}\right) + \gamma\frac{2}{3}z_{2,3} = a_{28}$
- $P(i_1, k_1|j_1, l_1) = (1 - \gamma)\frac{1}{3}z_{1,3} + \gamma\left(1 - \frac{2}{3}z_{2,3}\right) = a_{29}$
- $P(i_1, l_1|j_1, k_1) = (1 - \gamma)\frac{1}{3}z_{1,3} + \gamma\frac{1}{3}z_{2,3} = a_{30}$

$n = (1, 1, 0, 2)$

- Individuals $i_1 \in n_0$; $j_1 \in n_1$; $l_1, l_2 \in n_3$
- $P(l_1, l_2|i_1, j_1) = (1-\gamma)\left(1 - \frac{2}{3}z_{1,3} z_3\right) + \gamma\left(1 - \frac{2}{3}z_3\right) = a_{31}$
- $P(l_1, i_1|l_2, j_1) = (1 - \gamma)\frac{1}{3}z_{1,3} z_3 + \gamma\frac{1}{3}z_3 = a_{32}$
- $P(l_1, j_1|l_2, i_1) = (1 - \gamma)\frac{1}{3}z_{1,3} z_3 + \gamma\frac{1}{3}z_3 = a_{33}$

$n = (1, 2, 1, 0)$

- Individuals $i_1 \in n_0$; $j_1, j_2 \in n_1$; $k_1 \in n_2$
- $P(j_1, j_2|i_1, k_1) = (1-\gamma)\left(1 - \frac{2}{3}z_1\right) + \gamma\left(1 - \frac{2}{3}z_{2,3} z_{1,3} z_1\right) = a_{34}$
- $P(j_1, i_1|j_2, k_1) = (1 - \gamma)\frac{1}{3}z_1 + \gamma\frac{1}{3}z_{2,3} z_{1,3} z_1 = a_{35}$
- $P(j_1, k_1|j_2, i_1) = (1 - \gamma)\frac{1}{3}z_1 + \gamma\frac{1}{3}z_{2,3} z_{1,3} z_1 = a_{36}$

$n = (1, 2, 0, 1)$

- Individuals $i_1 \in n_0$; $j_1, j_2 \in n_1$; $l_1 \in n_3$
- $P(j_1, j_2|i_1, l_1) = (1 - \gamma)\left(1 - \frac{2}{3}z_1\right) + \gamma\left(1 - \frac{2}{3}z_{1,3} z_1\right) = a_{37}$
- $P(j_1, l_1|j_2, i_1) = (1 - \gamma)\frac{1}{3}z_1 + \gamma\frac{1}{3}z_{1,3} z_1 = a_{38}$
- $P(j_1, i_1|j_2, l_1) = (1 - \gamma)\frac{1}{3}z_1 + \gamma\frac{1}{3}z_{1,3} z_1 = a_{39}$

$n = (2, 0, 2, 0)$

- Individuals $i_1, i_2 \in n_0$; $k_1, k_2 \in n_2$
- $P(i_1, i_2|k_1, k_2) = (1-\gamma)^2\left(1 - \frac{2}{3}z_2 z_0 z_{0,1} z_{1,3} z_{2,3}\right) + 2\gamma(1-\gamma)\left(1 - \frac{2}{3}z_2 z_0\right) + \gamma^2\left(1 - \frac{2}{3}z_2 z_0 z_{0,2}\right) = a_{40}$
- $P(i_1, k_1|i_2, k_2) = (1 - \gamma)^2\frac{1}{3}z_2 z_0 z_{0,1} z_{1,3} z_{2,3} + 2\gamma(1 - \gamma)\frac{1}{3}z_2 z_0 + \gamma^2\frac{1}{3}z_2 z_0 z_{0,2} = a_{41}$
- $P(i_1, k_2|k_1, i_2) = (1 - \gamma)^2\frac{1}{3}z_2 z_0 z_{0,1} z_{1,3} z_{2,3} + 2\gamma(1 - \gamma)\frac{1}{3}z_2 z_0 + \gamma^2\frac{1}{3}z_2 z_0 z_{0,2} = a_{42}$

$n = (2, 0, 1, 1)$

- Individuals $i_1, i_2 \in n_0$; $k_1 \in n_2$; $l_1 \in n_3$
- $P(i_1, i_2|k_1, l_1) = (1 - \gamma)^2\left(1 - \frac{2}{3}z_0 z_{1,3} z_{0,1}\right) + 2\gamma(1 - \gamma)\left(1 - z_0 + \frac{1}{3}z_0 z_{2,3}\right) + \gamma^2\left(1 - \frac{2}{3}z_0 z_{0,2}\right) = a_{43}$
- $P(i_1, k_1|l_1, i_2) = (1 - \gamma)^2\frac{1}{3}z_0 z_{1,3} z_{0,1} + \gamma(1 - \gamma)z_0\left(1 - \frac{1}{3}z_{2,3}\right) + \gamma^2\frac{1}{3}z_0 z_{0,2} = a_{44}$
- $P(i_1, l_1|i_2, k_1) = (1 - \gamma)^2\frac{1}{3}z_0 z_{1,3} z_{0,1} + \gamma(1 - \gamma)z_0\left(1 - \frac{1}{3}z_{2,3}\right) + \gamma^2\frac{1}{3}z_0 z_{0,2} = a_{45}$

$n = (2, 0, 0, 2)$

- Individuals $i_1, i_2 \in n_0$; $l_1, l_2 \in n_3$
- $P(i_1, i_2|l_1, l_2) = (1 - \gamma)^2\left(1 - \frac{2}{3}z_3 z_0 z_{1,3} z_{0,1}\right) + 2\gamma(1 - \gamma)\left(1 - \frac{2}{3}z_3 z_0\right) + \gamma^2\left(1 - \frac{2}{3}z_3 z_0 z_{2,3} z_{0,2}\right) = a_{46}$

- $P(i_1, l_1|i_2, l_2) = (1-\gamma)^2 \frac{1}{3} z_3 z_0 z_{1,3} z_{0,1} + 2\gamma(1-\gamma)\frac{1}{3} z_3 z_0 + \gamma^2 \frac{1}{3} z_3 z_0 z_{2,3} z_{0,2} = a_{47}$
- $P(i_1, l_2|i_2, l_1) = (1-\gamma)^2 \frac{1}{3} z_3 z_0 z_{1,3} z_{0,1} + 2\gamma(1-\gamma)\frac{1}{3} z_3 z_0 + \gamma^2 \frac{1}{3} z_3 z_0 z_{2,3} z_{0,2} = a_{48}$

$n = (2, 1, 1, 0)$
- Individuals $i_1, i_2 \in n_0$; $j_1 \in n_1$; $k_1 \in n_2$
- $P(i_1, i_2|j_1, k_1) = (1-\gamma)^2 \left(1 - \frac{2}{3} z_0 z_{0,1}\right) + 2\gamma(1-\gamma)\left(1 - z_0 + \frac{1}{3} z_0 z_{2,3} z_{1,3}\right) + \gamma^2 \left(1 - \frac{2}{3} z_0 z_{0,2}\right) = a_{49}$
- $P(i_1, j_1|i_2, k_1) = 1 - \gamma)^2 \frac{1}{3} z_0 z_{0,1} + \gamma(1-\gamma) z_0 \left(1 - \frac{1}{3} z_{2,3} z_{1,3}\right) + \gamma^2 \frac{1}{3} z_0 z_{0,2} = a_{50}$
- $P(i_1, k_1|i_2, j_1) = 1 - \gamma)^2 \frac{1}{3} z_0 z_{0,1} + \gamma(1-\gamma) z_0 \left(1 - \frac{1}{3} z_{2,3} z_{1,3}\right) + \gamma^2 \frac{1}{3} z_0 z_{0,2} = a_{51}$

$n = (2, 1, 0, 1)$
- Individuals $i_1, i_2 \in n_0$; $j_1 \in n_1$; $l_1 \in n_3$
- $P(i_1, i_2|j_1, l_1) = (1-\gamma)^2 \left(1 - \frac{2}{3} z_0 z_{0,1}\right) + 2\gamma(1-\gamma)\left(1 - z_0 + \frac{1}{3} z_0 z_{1,3}\right) + \gamma^2 \left(1 - \frac{2}{3} z_0 z_{0,2} z_{2,3}\right) = a_{52}$
- $P(i_1, j_1|i_2, l_1) = (1-\gamma)^2 \frac{1}{3} z_0 z_{0,1} + \gamma(1-\gamma) z_0 \left(1 - \frac{1}{3} z_{1,3}\right) + \gamma^2 \frac{1}{3} z_0 z_{0,2} z_{2,3} = a_{53}$
- $P(i_1, l_1|i_2, j_1) = (1-\gamma)^2 \frac{1}{3} z_0 z_{0,1} + \gamma(1-\gamma) z_0 \left(1 - \frac{1}{3} z_{1,3}\right) + \gamma^2 \frac{1}{3} z_0 z_{0,2} z_{2,3} = a_{54}$

$n = (2, 2, 0, 0)$
- Individuals $i_1, i_2 \in n_0$; $j_1, j_2 \in n_1$;
- $P(i_1, i_2|j_1, j_2) = (1-\gamma)^2 \left(1 - \frac{2}{3} z_1 z_0 z_{0,1}\right) + 2\gamma(1-\gamma)\left(1 - \frac{2}{3} z_1 z_0\right) + \gamma^2 \left(1 - \frac{2}{3} z_1 z_0 z_{0,2} z_{2,3} z_{1,3}\right) = a_{55}$
- $P(i_1, j_1|i_2, j_2) = (1-\gamma)^2 \frac{1}{3} z_1 z_0 z_{0,1} + 2\gamma(1-\gamma)\frac{1}{3} z_1 z_0 + \gamma^2 \frac{1}{3} z_1 z_0 z_{0,2} z_{2,3} z_{1,3} = a_{56}$
- $P(i_1, j_2|i_2, j_1) = (1-\gamma)^2 \frac{1}{3} z_1 z_0 z_{0,1} + 2\gamma(1-\gamma)\frac{1}{3} z_1 z_0 + \gamma^2 \frac{1}{3} z_1 z_0 z_{0,2} z_{2,3} z_{1,3} = a_{57}$

## Phylogenetic invariants for $n$-taxon phylogenetic networks with one 4-node hybridization cycle

Below, we present the invariants for different networks $N$ all with one 4-cycle, but with different number of species on the clades $n_0, n_1, n_2, n_3$. For example, the network $N = 1112$ corresponds to a network with 5 species: one in $n_0$, one in $n_1$, one in $n_2$ and two in $n_3$. The number of species defines the number of CF formulas. For example, for 6 species, there are $\binom{6}{4} = 15$ 4-taxon subsets, each with 3 CF formulas. So, for 6 species, we have 45 CF formulas and thus, 45 CF values. However, we only want to focus on the 4-taxon subsets that involve the hybridization cycle. For the case of $N = 1112$, they are only 4: $(0, 1, 1, 2), (1, 0, 1, 2), (1, 1, 1, 1), (1, 1, 0, 2)$. Note that our table of CF formulas has 57 different CF formulas (and therefore, values). This discrepancy is due to the fact that the table is listing all possible CF formulas and we will have fewer formulas if we have less than 8 species.

For some examples, for computational restrictions, we had to include just a subset of the original CF equations to obtain the Gröbner basis in $a_i$. We denote these cases with "subset". All Macaulay2 scripts (and output) can be found in the GitHub repository: `https://github.com/solislemuslab/phylo-diamond.jl`.

- $N = 1112$
  1. $a_{32} - a_{33}$
  2. $a_{31} + 2a_{33} - 1$
  3. $a_{28} + a_{29} + a_{30} - 1$
  4. $a_{23} - a_{24}$
  5. $a_{22} + 2a_{24} - 1$
  6. $a_8 - a_9$
  7. $a_7 + 2a_9 - 1$
  8. $3a_9 * a_{30} + a_9 - a_{24} - a_{33}$
  9. $a_{24} * a_{29} + 2a_{24} * a_{30} + a_{29} * a_{33} - a_{30} * a_{33} - a_{33}$
  10. $3a_9 * a_{29} - 2a_9 + 2a_{24} - a_{33}$
- $N = 1121$
  1. $a_{28} + a_{29} + a_{30} - 1$
  2. $a_{26} - a_{27}$
  3. $a_{25} + 2a_{27} - 1$
  4. $a_{20} - a_{21}$
  5. $a_{19} + 2a_{21} - 1$
  6. $a_5 - a_6$
  7. $a_4 + 2a_6 - 1$
  8. $a_6 * a_{29} + 2a_6 * a_{30} - a_6 + a_{21} - a_{27}$
- $N = 1122$
  1. $a_{32} - a_{33}$
  2. $a_{31} + 2a_{33} - 1$
  3. $a_{28} + a_{29} + a_{30} - 1$
  4. $a_{26} - a_{27}$
  5. $a_{25} + 2a_{27} - 1$
  6. $a_{23} - a_{24}$
  7. $a_{22} + 2a_{24} - 1$
  8. $a_{20} - a_{21}$
  9. $a_{19} + 2a_{21} - 1$
  10. $a_8 - a_9$
  11. $a_7 + 2a_9 - 1$
  12. $a_5 - a_6$
  13. $a_4 + 2a_6 - 1$
  14. $a_2 - a_3$
  15. $a_1 + 2a_3 - 1$
  16. $3a_9 * a_{30} + a_9 - a_{24} - a_{33}$
  17. $a_{24} * a_{29} + 2a_{24} * a_{30} + a_{29} * a_{33} - a_{30} * a_{33} - a_{33}$
  18. $3a_9 * a_{29} - 2a_9 + 2a_{24} - a_{33}$
  19. $a_6 * a_{29} + 2a_6 * a_{30} - a_6 + a_{21} - a_{27}$
  20. $a_3 * a_{29} + 2a_3 * a_{30} - 3a_6 * a_{33}$
  21. $3a_6 * a_{24} - 3a_3 * a_{30} + 3a_6 * a_{33} - a_3$
  22. $3a_9 * a_{21} - 3a_9 * a_{27} + 3a_6 * a_{33} - a_3$
  23. $3a_6 * a_9 - a_3$
- $N = 1211$
  1. $a_{38} - a_{39}$
  2. $a_{37} + 2a_{39} - 1$
  3. $a_{35} - a_{36}$
  4. $a_{34} + 2a_{36} - 1$
  5. $a_{28} + a_{29} + a_{30} - 1$

6. $a_{14} - a_{15}$
7. $a_{13} + 2a_{15} - 1$
8. $a_{15} * a_{29} - a_{15} * a_{30} + a_{36} - a_{39}$

- $N = 1212$

1. $a_{38} - a_{39}$
2. $a_{37} + 2a_{39} - 1$
3. $a_{35} - a_{36}$
4. $a_{34} + 2a_{36} - 1$
5. $a_{32} - a_{33}$
6. $a_{31} + 2a_{33} - 1$
7. $a_{28} + a_{29} + a_{30} - 1$
8. $a_{23} - a_{24}$
9. $a_{22} + 2a_{24} - 1$
10. $a_{17} - a_{18}$
11. $a_{16} + 2a_{18} - 1$
12. $a_{14} - a_{15}$
13. $a_{13} + 2a_{15} - 1$
14. $a_8 - a_9$
15. $a_7 + 2a_9 - 1$
16. $a_{18} * a_{30} - a_{15} * a_{33} - a_9 * a_{36} + a_9 * a_{39}$
17. $3a_9 * a_{30} + a_9 - a_{24} - a_{33}$
18. $a_{24} * a_{29} + 2a_{24} * a_{30} + a_{29} * a_{33} - a_{30} * a_{33} - a_{33}$
19. $a_{18} * a_{29} - a_{15} * a_{33} + 2a_9 * a_{36} - 2a_9 * a_{39}$
20. $a_{15} * a_{29} - a_{15} * a_{30} + a_{36} - a_{39}$
21. $3a_9 * a_{29} - 2a_9 + 2a_{24} - a_{33}$
22. $3a_{15} * a_{24} - 3a_9 * a_{36} + 3a_9 * a_{39} - a_{18}$
23. $3a_9 * a_{15} - a_{18}$

- $N = 1221$

1. $a_{38} - a_{39}$
2. $a_{37} + 2a_{39} - 1$
3. $a_{35} - a_{36}$
4. $a_{34} + 2a_{36} - 1$
5. $a_{28} + a_{29} + a_{30} - 1$
6. $a_{26} - a_{27}$
7. $a_{25} + 2a_{27} - 1$
8. $a_{20} - a_{21}$
9. $a_{19} + 2a_{21} - 1$
10. $a_{14} - a_{15}$
11. $a_{13} + 2a_{15} - 1$
12. $a_{11} - a_{12}$
13. $a_{10} + 2a_{12} - 1$
14. $a_5 - a_6$
15. $a_4 + 2a_6 - 1$
16. $a_{15} * a_{29} - a_{15} * a_{30} + a_{36} - a_{39}$
17. $a_{12} * a_{29} - a_{12} * a_{30} + 3a_6 * a_{36} - 3a_6 * a_{39}$
18. $a_6 * a_{29} + 2a_6 * a_{30} - a_6 + a_{21} - a_{27}$
19. $3a_{15}*a_{21} - 3a_{15}*a_{27} + 3a_{12}*a_{30} - 3a_6*a_{36} + 3a_6*a_{39} - a_{12}$
20. $3a_6 * a_{15} - a_{12}$
21. $a_{21} * a_{29} * a_{36} + 2a_{21} * a_{30} * a_{36} - a_{27} * a_{29} * a_{39} + a_{27} * a_{30} * a_{39} - a_{15} * a_{27} + a_{12} * a_{30} - a_6 * a_{36} - a_{21} * a_{36} - a_{27} * a_{36} + 2a_{21} * a_{39}$

- $N = 1222$

1. $a_{38} - a_{39}$
2. $a_{37} + 2a_{39} - 1$
3. $a_{35} - a_{36}$
4. $a_{34} + 2a_{36} - 1$
5. $a_{32} - a_{33}$
6. $a_{31} + 2a_{33} - 1$
7. $a_{28} + a_{29} + a_{30} - 1$
8. $a_{26} - a_{27}$
9. $a_{25} + 2a_{27} - 1$
10. $a_{23} - a_{24}$
11. $a_{22} + 2a_{24} - 1$
12. $a_{20} - a_{21}$
13. $a_{19} + 2a_{21} - 1$
14. $a_{17} - a_{18}$
15. $a_{16} + 2a_{18} - 1$
16. $a_{14} - a_{15}$
17. $a_{13} + 2a_{15} - 1$
18. $a_{11} - a_{12}$
19. $a_{10} + 2a_{12} - 1$
20. $a_8 - a_9$
21. $a_7 + 2a_9 - 1$
22. $a_5 - a_6$
23. $a_4 + 2a_6 - 1$
24. $a_2 - a_3$
25. $a_1 + 2a_3 - 1$
26. $a_{18} * a_{30} - a_{15} * a_{33} - a_9 * a_{36} + a_9 * a_{39}$
27. $3a_9 * a_{30} + a_9 - a_{24} - a_{33}$
28. $a_{24} * a_{29} + 2a_{24} * a_{30} + a_{29} * a_{33} - a_{30} * a_{33} - a_{33}$
29. $a_{18} * a_{29} - a_{15} * a_{33} + 2a_9 * a_{36} - 2a_9 * a_{39}$
30. $a_{15} * a_{29} - a_{15} * a_{30} + a_{36} - a_{39}$
31. $a_{12} * a_{29} - a_{12} * a_{30} + 3a_6 * a_{36} - 3a_6 * a_{39}$
32. $3a_9 * a_{29} - 2a_9 + 2a_{24} - a_{33}$
33. $a_6 * a_{29} + 2a_6 * a_{30} - a_6 + a_{21} - a_{27}$
34. $a_3 * a_{29} + 2a_3 * a_{30} - 3a_6 * a_{33}$
35. $3a_{15} * a_{24} - 3a_9 * a_{36} + 3a_9 * a_{39} - a_{18}$
36. $3a_6 * a_{24} - 3a_3 * a_{30} + 3a_6 * a_{33} - a_3$
37. $a_{18}*a_{21} - a_{12}*a_{24} - a_{18}*a_{27} + a_{12}*a_{33} + a_3*a_{36} - a_3*a_{39}$
38. $3a_{15}*a_{21} - 3a_{15}*a_{27} + 3a_{12}*a_{30} - 3a_6*a_{36} + 3a_6*a_{39} - a_{12}$
39. $3a_9 * a_{21} - 3a_9 * a_{27} + 3a_6 * a_{33} - a_3$
40. $a_6 * a_{18} - a_{12} * a_{24} + a_3 * a_{36} - a_3 * a_{39}$
41. $3a_9 * a_{15} - a_{18}$
42. $3a_6 * a_{15} - a_{12}$
43. $a_3 * a_{15} - a_{12} * a_{24} + a_3 * a_{36} - a_3 * a_{39}$
44. $a_9 * a_{12} - a_{12} * a_{24} + a_3 * a_{36} - a_3 * a_{39}$
45. $3a_6 * a_9 - a_3$
46. $a_{21} * a_{29} * a_{36} + 2a_{21} * a_{30} * a_{36} - a_{27} * a_{29} * a_{39} + a_{27} * a_{30} * a_{39} - a_{15} * a_{27} + a_{12} * a_{30} - a_6 * a_{36} - a_{21} * a_{36} - a_{27} * a_{36} + 2a_{21} * a_{39}$
47. $6a_9 * a_{27} * a_{36} - 3a_6 * a_{33} * a_{36} - 3a_{21} * a_{33} * a_{36} - 3a_9 * a_{27} * a_{39} - 3a_{24} * a_{27} * a_{39} + 6a_6 * a_{33} * a_{39} + a_{18} * a_{27} - a_{12} * a_{33} + a_3 * a_{36} - a_3 * a_{39}$

- $N = 2111$

1. $a_{53} - a_{54}$
2. $a_{52} + 2a_{54} - 1$
3. $a_{50} - a_{51}$
4. $a_{49} + 2a_{51} - 1$
5. $a_{44} - a_{45}$
6. $a_{43} + 2a_{45} - 1$
7. $a_{28} + a_{29} + a_{30} - 1$

- $N = 2112$

1. $a_{53} - a_{54}$
2. $a_{52} + 2a_{54} - 1$
3. $a_{50} - a_{51}$
4. $a_{49} + 2a_{51} - 1$
5. $a_{47} - a_{48}$
6. $a_{46} + 2a_{48} - 1$
7. $a_{44} - a_{45}$
8. $a_{43} + 2a_{45} - 1$
9. $a_{32} - a_{33}$

10. $a_{31} + 2a_{33} - 1$
11. $a_{28} + a_{29} + a_{30} - 1$
12. $a_{23} - a_{24}$
13. $a_{22} + 2a_{24} - 1$
14. $a_8 - a_9$
15. $a_7 + 2a_9 - 1$
16. $3a_9 * a_{30} + a_9 - a_{24} - a_{33}$
17. $a_{24} * a_{29} + 2a_{24} * a_{30} + a_{29} * a_{33} - a_{30} * a_{33} - a_{33}$
18. $3a_9 * a_{29} - 2a_9 + 2a_{24} - a_{33}$

- $N = 2121$

1. $a_4 + 2a_6 - 1$
2. $a_5 - a_6$
3. $a_{28} + a_{29} + a_{30} - 1$
4. $a_{19} + 2a_{21} - 1$
5. $a_{20} - a_{21}$
6. $a_{25} + 2a_{27} - 1$
7. $a_{26} - a_{27}$
8. $a_{40} + 2a_{42} - 1$
9. $a_{41} - a_{42}$
10. $a_{43} + 2a_{45} - 1$
11. $a_{44} - a_{45}$
12. $a_{49} + 2a_{51} - 1$
13. $a_{50} - a_{51}$
14. $a_{52} + 2a_{54} - 1$
15. $a_{53} - a_{54}$
16. $a_5 * a_{29} + 2a_5 * a_{30} - a_5 + a_{20} - a_{26}$
17. $2a_{20} * a_{29}^3 a_{41} + a_{26} * a_{29}^3 a_{41} + 3a_{20} * a_{29}^2 a_{30} * a_{41} - 3a_{20} * a_{29} * a_{30}^2 a_{41} - 3a_{26} * a_{29} * a_{30}^2 a_{41} - 2a_{20} * a_{30}^3 a_{41} + 2a_{26} * a_{30}^3 a_{41} - 3a_{20} * a_{26} * a_{29}^2 a_{44} - 3a_{26}^2 a_{29}^2 a_{44} - 3a_{20} * a_{26} * a_{29} * a_{30} * a_{44} + 6a_{26}^2 a_{29} * a_{30} * a_{44} + 6a_{20} * a_{26} * a_{30}^2 a_{44} - 3a_{26}^2 a_{30}^2 a_{44} - 6a_{20}^2 a_{29}^2 a_{50} + 3a_{20} * a_{26} * a_{29}^2 a_{50} - 15a_{20}^2 a_{29} * a_{30} * a_{50} - 6a_{20} * a_{26} * a_{29} * a_{30} * a_{50} - 6a_{20}^2 a_{30}^2 a_{50} + 3a_{20} * a_{26} * a_{30}^2 a_{50} + 3a_{20} * a_{26} * a_{29}^2 a_{53} + 3a_{20} * a_{26} * a_{29} * a_{30} * a_{53} - 6a_{20} * a_{26} * a_{30}^2 a_{53} - 4a_{20} * a_{29}^2 a_{41} - 2a_{26} * a_{29}^2 a_{41} - a_{20} * a_{29} * a_{30} * a_{41} + a_{26} * a_{29} * a_{30} * a_{41} + 5a_{20} * a_{30}^2 a_{41} + a_{26} * a_{30}^2 a_{41} + 3a_{20}^2 a_{29} * a_{44} + 6a_{20} * a_{26} * a_{29} * a_{44} + 3a_{26}^2 a_{29} * a_{44} + 6a_{20}^2 a_{30} * a_{44} + 18a_5 * a_{26} * a_{30} * a_{44} - 6a_{20} * a_{26} * a_{30} * a_{44} - 3a_{26}^2 a_{30} * a_{44} + 6a_{20} * a_{26} * a_{29} * a_{50} - 9a_5 * a_{20} * a_{30} * a_{50} + 9a_{20}^2 a_{30} * a_{50} - 9a_5 * a_{26} * a_{30} * a_{50} + 12a_{20} * a_{26} * a_{30} * a_{50} - 3a_{20}^2 a_{29} * a_{53} - 3a_{26}^2 a_{29} * a_{53} - 6a_{20}^2 a_{30} * a_{53} + 3a_{26}^2 a_{30} * a_{53} + 3a_{26} * a_{29} * a_{41} - 3a_{26} * a_{30} * a_{41} - 3a_5^2 a_{44} + 3a_5 * a_{20} * a_{44} - 3a_5 * a_{26} * a_{44} - 6a_{26}^2 a_{44} + 3a_5^2 a_{50} - 6a_5 * a_{20} * a_{50} + 3a_{20}^2 a_{50} + 6a_5 * a_{26} * a_{50} - 6a_{20} * a_{26} * a_{50} + 3a_5 * a_{20} * a_{53} - 3a_{20}^2 a_{53} - 3a_5 * a_{26} * a_{53} + 6a_{20} * a_{26} * a_{53}$

- $N = 2211$

1. $a_{13} + 2a_{15} - 1$
2. $a_{14} - a_{15}$
3. $a_{28} + a_{29} + a_{30} - 1$
4. $a_{34} + 2a_{36} - 1$
5. $a_{35} - a_{36}$
6. $a_{37} + 2a_{39} - 1$
7. $a_{38} - a_{39}$
8. $a_{43} + 2a_{45} - 1$
9. $a_{44} - a_{45}$
10. $a_{49} + 2a_{51} - 1$
11. $a_{50} - a_{51}$
12. $a_{52} + 2a_{54} - 1$
13. $a_{53} - a_{54}$
14. $a_{55} + 2a_{57} - 1$
15. $a_{56} - a_{57}$
16. $a_{14} * a_{29} - a_{14} * a_{30} + a_{35} - a_{38}$
17. $3a_{29}^2 a_{35} * a_{38} * a_{44} + 3a_{29} * a_{30} * a_{35} * a_{38} * a_{44} - 6a_{30}^2 a_{35} * a_{38} * a_{44} + 3a_{29}^2 a_{35} * a_{38} * a_{50} + 12a_{29} * a_{30} * a_{35} * a_{38} * a_{50} + 12a_{30}^2 a_{35} * a_{38} * a_{50} - 6a_{29}^2 a_{38}^2 a_{50} + 3a_{29} * a_{30} * a_{38}^2 a_{50} + 3a_{30}^2 a_{38}^2 a_{50} - 3a_{29}^2 a_{35}^2 a_{53} - 12a_{29} * a_{30} * a_{35}^2 a_{53} - 12a_{30}^2 a_{35}^2 a_{53} - 3a_{29}^2 a_{35} * a_{38} * a_{53} - 3a_{29} * a_{30} * a_{35} * a_{38} * a_{53} + 6a_{30}^2 a_{35} * a_{38} * a_{53} - a_{29}^3 a_{35} * a_{56} - 3a_{29}^2 a_{30} * a_{35} * a_{56} + 4a_{30}^3 a_{35} * a_{56} - 2a_{29}^3 a_{38} * a_{56} - 3a_{29}^2 a_{30} * a_{38} * a_{56} + 3a_{29} * a_{30}^2 a_{38} * a_{56} + 2a_{30}^3 a_{38} * a_{56} + 3a_{29} * a_{35}^2 a_{44} + 6a_{30} * a_{35}^2 a_{44} - 6a_{29} * a_{35} * a_{38} * a_{44} - 3a_{30} * a_{35} * a_{38} * a_{44} + 3a_{29} * a_{38}^2 a_{44} - 3a_{30} * a_{38}^2 a_{44} - 9a_{14} * a_{30} * a_{35} * a_{50} - 9a_{14} * a_{30} * a_{38} * a_{50} - 12a_{29} * a_{35} * a_{38} * a_{50} - 6a_{30} * a_{35} * a_{38} * a_{50} + 12a_{29} * a_{38}^2 a_{50} + 6a_{30} * a_{38}^2 a_{50} + 18a_{14} * a_{30} * a_{35} * a_{53} + 3a_{29} * a_{35}^2 a_{53} + 6a_{30} * a_{35}^2 a_{53} - 9a_{30} * a_{35} * a_{38} * a_{53} - 3a_{29} * a_{38}^2 a_{53} + 3a_{30} * a_{38}^2 a_{53} + a_{29}^2 a_{35} * a_{56} + a_{29} * a_{30} * a_{35} * a_{56} - 2a_{30}^2 a_{35} * a_{56} + 2a_{29}^2 a_{38} * a_{56} - a_{29} * a_{30} * a_{38} * a_{56} - a_{30}^2 a_{38} * a_{56} - 3a_{14} * a_{35} * a_{44} - 3a_{35}^2 a_{44} + 3a_{14} * a_{38} * a_{44} + 9a_{35} * a_{38} * a_{44} - 6a_{38}^2 a_{44} + 3a_{14}^2 a_{50} + 6a_{14} * a_{35} * a_{50} - 6a_{14} * a_{38} * a_{50} + 3a_{35} * a_{38} * a_{50} - 3a_{38}^2 a_{50} - 3a_{14}^2 a_{53} - 3a_{14} * a_{35} * a_{53} - 6a_{35}^2 a_{53} + 3a_{14} * a_{38} * a_{53} + 3a_{35} * a_{38} * a_{53} + 3a_{38}^2 a_{53} - 2a_{29} * a_{35} * a_{56} - 4a_{30} * a_{35} * a_{56} + 2a_{29} * a_{38} * a_{56} + 4a_{30} * a_{38} * a_{56} + 2a_{35} * a_{56} - 2a_{38} * a_{56}$

- $N = 2212$

1. $a_{16} + 2a_{18} - 1$
2. $a_{17} - a_{18}$
3. $a_{28} + a_{29} + a_{30} - 1$
4. $a_{13} + 2a_{15} - 1$
5. $a_{14} - a_{15}$
6. $a_{31} + 2a_{33} - 1$
7. $a_{32} - a_{33}$
8. $a_7 + 2a_9 - 1$
9. $a_8 - a_9$
10. $a_{34} + 2a_{36} - 1$
11. $a_{35} - a_{36}$
12. $a_{37} + 2a_{39} - 1$
13. $a_{38} - a_{39}$
14. $a_{22} + 2a_{24} - 1$
15. $a_{23} - a_{24}$
16. $a_{43} + 2a_{45} - 1$
17. $a_{44} - a_{45}$
18. $a_{46} + 2a_{48} - 1$
19. $a_{47} - a_{48}$
20. $a_{49} + 2a_{51} - 1$
21. $a_{50} - a_{51}$
22. $a_{52} + 2a_{54} - 1$
23. $a_{53} - a_{54}$
24. $a_{55} + 2a_{57} - 1$
25. $a_{56} - a_{57}$
26. $a_{17} * a_{30} - a_{14} * a_{32} - a_8 * a_{35} + a_8 * a_{38}$
27. $3a_8 * a_{30} + a_8 - a_{23} - a_{32}$
28. $a_{23} * a_{29} + 2a_{23} * a_{30} + a_{29} * a_{32} - a_{30} * a_{32} - a_{32}$
29. $a_{17} * a_{29} - a_{14} * a_{32} + 2a_8 * a_{35} - 2a_8 * a_{38}$
30. $a_{14} * a_{29} - a_{14} * a_{30} + a_{35} - a_{38}$
31. $3a_8 * a_{29} - 2a_8 + 2a_{23} - a_{32}$
32. $3a_{14} * a_{23} - 3a_8 * a_{35} + 3a_8 * a_{38} - a_{17}$
33. $3a_8 * a_{14} - a_{17}$
34. $3a_{32} * a_{38} * a_{44} - 2a_{29} * a_{38} * a_{47} - a_{30} * a_{38} * a_{47} - 3a_{32} * a_{38} * a_{50} + 3a_{32} * a_{35} * a_{53} + 3a_8 * a_{38} * a_{53} - 3a_{23} * a_{38} * a_{53} + a_{29} * a_{32} * a_{56} - a_{30} * a_{32} * a_{56} - a_{17} * a_{44} + a_{14} * a_{47} - a_{35} * a_{47} + a_{38} * a_{47} + a_{17} * a_{50} - a_{17} * a_{53} - a_8 * a_{56} + a_{23} * a_{56}$
35. $3a_8 * a_{35} * a_{44} - 3a_{32} * a_{35} * a_{44} - 3a_8 * a_{38} * a_{44} + a_{29} * a_{35} * a_{47} + 2a_{30} * a_{35} * a_{47} + 3a_8 * a_{35} * a_{50} - 3a_{23} * a_{38} *$

$a_{50} + a_{29} * a_{32} * a_{56} - a_{30} * a_{32} * a_{56} + a_{17} * a_{44} - a_{14} * a_{47} - a_{35} * a_{47} + a_{38} * a_{47} - a_8 * a_{56} + a_{23} * a_{56}$

36. $3a_{17} * a_{32} * a_{44}^2 - 6a_{14} * a_{32} * a_{44} * a_{47} + 3a_{32} * a_{35} * a_{44} * a_{47} + 3a_{14} * a_{30} * a_{47}^2 - a_{29} * a_{35} * a_{47}^2 - 2a_{30} * a_{35} * a_{47}^2 - 3a_8 * a_{17} * a_{44} * a_{50} - 3a_{17} * a_{32} * a_{44} * a_{50} + 3a_{14} * a_{32} * a_{47} * a_{50} - 3a_8 * a_{35} * a_{47} * a_{50} + 3a_{23} * a_{38} * a_{47} * a_{50} + 3a_8 * a_{17} * a_{50}^2 - 3a_{17} * a_{23} * a_{44} * a_{53} + 3a_8 * a_{35} * a_{47} * a_{53} - 3a_8 * a_{38} * a_{47} * a_{53} - 3a_{17} * a_{23} * a_{50} * a_{53} + 6a_8 * a_{32} * a_{44} * a_{56} - a_{29} * a_{32} * a_{47} * a_{56} + a_{30} * a_{32} * a_{47} * a_{56} - 3a_8^2 * a_{50} * a_{56} + 3a_8 * a_{23} * a_{50} * a_{56} - 3a_8 * a_{32} * a_{50} * a_{56} + 3a_8^2 * a_{53} * a_{56} - 3a_8 * a_{23} * a_{53} * a_{56} + 3a_8 * a_{32} * a_{53} * a_{56} - a_{35} * a_{47}^2 + a_{38} * a_{47}^2 + a_{17} * a_{47} * a_{50} + a_{17} * a_{47} * a_{53} - 2a_{32} * a_{47} * a_{56}$

37. $9a_8 * a_{23} * a_{38} * a_{44} * a_{50} - 9a_8 * a_{23} * a_{38} * a_{50}^2 + 9a_{23} * a_{32} * a_{35} * a_{44} * a_{53} + 9a_8 * a_{23} * a_{38} * a_{44} * a_{53} + 3a_{29} * a_{32} * a_{35} * a_{47} * a_{53} - 3a_{30} * a_{32} * a_{35} * a_{47} * a_{53} + 9a_{23}^2 * a_{38} * a_{50} * a_{53} + 9a_{23} * a_{30} * a_{32} * a_{53} * a_{56} + 3a_{29} * a_{32}^2 * a_{53} * a_{56} - 3a_{30} * a_{32}^2 * a_{53} * a_{56} - 3a_{17} * a_{23} * a_{44}^2 - 3a_{23} * a_{35} * a_{44} * a_{47} + 6a_{32} * a_{35} * a_{44} * a_{47} - 3a_{29} * a_{35} * a_{47}^2 - 3a_{30} * a_{35} * a_{47}^2 + 3a_{17} * a_{23} * a_{44} * a_{50} - 9a_8 * a_{35} * a_{47} * a_{50} + 3a_8 * a_{38} * a_{47} * a_{50} + 3a_{23} * a_{38} * a_{47} * a_{50} - 3a_{17} * a_{23} * a_{44} * a_{53} + 3a_8 * a_{35} * a_{47} * a_{53} - 3a_{32} * a_{35} * a_{47} * a_{53} - 3a_8 * a_{38} * a_{47} * a_{53} - 3a_{23} * a_{38} * a_{47} * a_{53} - 6a_8 * a_{23} * a_{44} * a_{56} - 3a_{23} * a_{30} * a_{47} * a_{56} - 3a_{29} * a_{32} * a_{47} * a_{56} + 3a_{30} * a_{32} * a_{47} * a_{56} + 6a_8 * a_{23} * a_{50} * a_{56} - 3a_{23}^2 * a_{50} * a_{56} - 3a_8 * a_{23} * a_{53} * a_{56} - 3a_{32}^2 * a_{53} * a_{56} + a_{14} * a_{47}^2 + 2a_{35} * a_{47}^2 - a_{38} * a_{47}^2 - a_{17} * a_{47} * a_{50} + a_{17} * a_{47} * a_{53} + 2a_8 * a_{47} * a_{56} + a_{32} * a_{47} * a_{56}$

38. $9a_{23} * a_{32} * a_{35} * a_{44}^2 + 9a_{23} * a_{30} * a_{35} * a_{44} * a_{47} + a_{29}^2 * a_{35} * a_{47}^2 + 4a_{29} * a_{30} * a_{35} * a_{47}^2 - 5a_{30}^2 * a_{35} * a_{47}^2 - 18a_{23} * a_{32} * a_{35} * a_{44} * a_{50} - 6a_{29} * a_{32} * a_{35} * a_{47} * a_{50} + 6a_{30} * a_{32} * a_{35} * a_{47} * a_{50} + 27a_{23} * a_{30} * a_{38} * a_{47} * a_{50} + 9a_{29} * a_{32} * a_{38} * a_{47} * a_{50} - 9a_{30} * a_{32} * a_{38} * a_{47} * a_{50} + 18a_8 * a_{23} * a_{35} * a_{50}^2 - 9a_8 * a_{23} * a_{38} * a_{50}^2 - 9a_{23}^2 * a_{38} * a_{50}^2 - 9a_{23}^2 * a_{35} * a_{44} * a_{53} + 9a_{23} * a_{32} * a_{35} * a_{44} * a_{53} + 9a_8 * a_{23} * a_{38} * a_{44} * a_{53} + 9a_{23} * a_{30} * a_{35} * a_{47} * a_{53} + 6a_{29} * a_{32} * a_{35} * a_{47} * a_{53} - 6a_{30} * a_{32} * a_{35} * a_{47} * a_{53} - 9a_{23}^2 * a_{35} * a_{50} * a_{53} + 9a_{23}^2 * a_{38} * a_{50} * a_{53} + 9a_{23} * a_{30} * a_{32} * a_{44} * a_{56} + 3a_{29} * a_{32}^2 * a_{44} * a_{56} - 3a_{30} * a_{32}^2 * a_{44} * a_{56} + 9a_{23} * a_{30}^2 * a_{47} * a_{56} + a_{29}^2 * a_{32} * a_{47} * a_{56} + a_{29} * a_{30} * a_{32} * a_{47} * a_{56} - 2a_{30}^2 * a_{32} * a_{47} * a_{56} + 9a_{23}^2 * a_{30} * a_{50} * a_{56} - 27a_{23} * a_{30} * a_{32} * a_{50} * a_{56} - 9a_{29} * a_{32}^2 * a_{50} * a_{56} + 9a_{30} * a_{32}^2 * a_{50} * a_{56} - 9a_{23}^2 * a_{30} * a_{53} * a_{56} + 18a_{23} * a_{30} * a_{32} * a_{53} * a_{56} + 6a_{29} * a_{32}^2 * a_{53} * a_{56} - 6a_{30} * a_{32}^2 * a_{53} * a_{56} - 3a_{17} * a_{23} * a_{44}^2 - 9a_{23} * a_{35} * a_{44} * a_{47} - 3a_{32} * a_{35} * a_{44} * a_{47} + 9a_{23} * a_{38} * a_{44} * a_{47} - 3a_{29} * a_{35} * a_{47}^2 + 3a_{29} * a_{38} * a_{47}^2 - 3a_{30} * a_{38} * a_{47}^2 + 6a_{17} * a_{23} * a_{44} * a_{50} - 3a_8 * a_{35} * a_{47} * a_{50} - 9a_{23} * a_{35} * a_{47} * a_{50} + 6a_{32} * a_{35} * a_{47} * a_{50} + 6a_8 * a_{38} * a_{47} * a_{50} + 6a_{23} * a_{38} * a_{47} * a_{50} - 9a_{32} * a_{38} * a_{47} * a_{50} - 3a_{17} * a_{23} * a_{44} * a_{53} + 3a_8 * a_{35} * a_{47} * a_{53} + 3a_{23} * a_{35} * a_{47} * a_{53} - 6a_{32} * a_{35} * a_{47} * a_{53} - 3a_8 * a_{38} * a_{47} * a_{53} - 3a_{23} * a_{38} * a_{47} * a_{53} - 3a_8 * a_{23} * a_{44} * a_{56} + 3a_{23}^2 * a_{44} * a_{56} - 3a_{32}^2 * a_{44} * a_{56} - 12a_{23} * a_{30} * a_{47} * a_{56} - 4a_{29} * a_{32} * a_{47} * a_{56} + a_{30} * a_{32} * a_{47} * a_{56} - 3a_{23} * a_{32} * a_{50} * a_{56} + 9a_{32}^2 * a_{50} * a_{56} - 3a_8 * a_{23} * a_{53} * a_{56} + 3a_{23}^2 * a_{53} * a_{56} + 3a_{23} * a_{32} * a_{53} * a_{56} - 6a_{32}^2 * a_{53} * a_{56} + a_{14} * a_{47}^2 + 4a_{35} * a_{47}^2 - 4a_{38} * a_{47}^2 - 2a_{17} * a_{47} * a_{50} + a_{17} * a_{47} * a_{53} - a_8 * a_{47} * a_{56} + a_{23} * a_{47} * a_{56} + 4a_{32} * a_{47} * a_{56}$

39. $3a_{14} * a_{17}^2 * a_{44}^2 - 6a_{14}^2 * a_{17} * a_{44} * a_{47} + 3a_{14} * a_{17} * a_{35} * a_{44} * a_{47} + 3a_{14}^3 * a_{47}^2 - 3a_{14}^2 * a_{35} * a_{47}^2 - 3a_{14} * a_{17}^2 * a_{44} * a_{50} - 3a_{17}^2 * a_{38} * a_{44} * a_{50} + 3a_{14}^2 * a_{17} * a_{47} * a_{50} + 3a_{14} * a_{17} * a_{38} * a_{47} * a_{50} + 3a_{17}^2 * a_{38} * a_{50}^2 - 3a_{17}^2 * a_{35} * a_{44} * a_{53} + 3a_{14} * a_{17} * a_{35} * a_{47} * a_{53} - 3a_{17}^2 * a_{35} * a_{50} * a_{53} + 3a_8 * a_{17} * a_{35} * a_{50} * a_{56} - 3a_8 * a_{17} * a_{38} * a_{50} * a_{56} - 3a_8 * a_{17} * a_{35} * a_{53} * a_{56} + 3a_8 * a_{17} * a_{38} * a_{53} * a_{56} + 2a_{17}^2 * a_{44} * a_{56} - 2a_{14} * a_{17} * a_{47} * a_{56} + a_{17} * a_{35} * a_{47} * a_{56} - a_{17} * a_{38} * a_{47} * a_{56} - a_{17}^2 * a_{50} * a_{56} + a_{17}^2 * a_{53} * a_{56}$

40. $3a_{29}^2 * a_{35} * a_{38} * a_{44} + 3a_{29} * a_{30} * a_{35} * a_{38} * a_{44} - 6a_{30}^2 * a_{35} * a_{38} * a_{44} + 3a_{29}^2 * a_{35} * a_{38} * a_{50} + 12a_{29} * a_{30} * a_{35} * a_{38} * a_{50} + 12a_{30}^2 * a_{35} * a_{38} * a_{50} - 6a_{29}^2 * a_{38}^2 * a_{50} + 3a_{29} * a_{30} * a_{38}^2 * a_{50} + 3a_{30}^2 * a_{38}^2 * a_{50} - 3a_{29}^2 * a_{35}^2 * a_{53} - 12a_{29} * a_{30} * a_{35}^2 * a_{53} - 12a_{30}^2 * a_{35}^2 * a_{53} - 3a_{29}^2 * a_{35} * a_{38} * a_{53} - 3a_{29} * a_{30} * a_{35} * a_{38} * a_{53} + 6a_{30}^2 * a_{35} * a_{38} * a_{53} - a_{29}^3 * a_{35} * a_{56} - 3a_{29}^2 * a_{30} * a_{35} * a_{56} + 4a_{30}^3 * a_{35} * a_{56} - 2a_{29}^3 * a_{38} * a_{56} - 3a_{29}^2 * a_{30} * a_{38} * a_{56} + 3a_{29} * a_{30}^2 * a_{38} * a_{56} + 2a_{30}^3 * a_{38} * a_{56} + 3a_{29} * a_{35}^2 * a_{44} + 6a_{30} * a_{35}^2 * a_{44} - 6a_{29} * a_{35} * a_{38} * a_{44} - 3a_{30} * a_{35} * a_{38} * a_{44} + 3a_{29} * a_{38}^2 * a_{44} - 3a_{30} * a_{38}^2 * a_{44} - 9a_{14} * a_{30} * a_{35} * a_{50} - 9a_{14} * a_{30} * a_{38} * a_{50} - 12a_{29} * a_{35} * a_{38} * a_{50} - 6a_{30} * a_{35} * a_{38} * a_{50} + 12a_{29} * a_{38}^2 * a_{50} + 6a_{30} * a_{38}^2 * a_{50} + 18a_{14} * a_{30} * a_{35} * a_{53} + 3a_{29} * a_{35}^2 * a_{53} + 6a_{30} * a_{35}^2 * a_{53} - 9a_{30} * a_{35} * a_{38} * a_{53} - 3a_{29} * a_{38}^2 * a_{53} + 3a_{30} * a_{38}^2 * a_{53} + a_{29}^2 * a_{35} * a_{56} + a_{29} * a_{30} * a_{35} * a_{56} - 2a_{30}^2 * a_{35} * a_{56} + 2a_{29}^2 * a_{38} * a_{56} - a_{29} * a_{30} * a_{38} * a_{56} - a_{30}^2 * a_{38} * a_{56} - 3a_{14} * a_{35} * a_{44} - 3a_{35}^2 * a_{44} + 3a_{14} * a_{38} * a_{44} + 9a_{35} * a_{38} * a_{44} - 6a_{38}^2 * a_{44} + 3a_{14}^2 * a_{50} + 6a_{14} * a_{35} * a_{50} - 6a_{14} * a_{38} * a_{50} + 3a_{35} * a_{38} * a_{50} - 3a_{38}^2 * a_{50} - 3a_{14}^2 * a_{53} - 3a_{14} * a_{35} * a_{53} - 6a_{35}^2 * a_{53} + 3a_{14} * a_{38} * a_{53} + 3a_{35} * a_{38} * a_{53} + 3a_{38}^2 * a_{53} - 2a_{29} * a_{35} * a_{56} - 4a_{30} * a_{35} * a_{56} + 2a_{29} * a_{38} * a_{56} + 4a_{30} * a_{38} * a_{56} + 2a_{35} * a_{56} - 2a_{38} * a_{56}$

- $N = 2122$ (subset)

1. $a_7 + 2a_9 - 1$
2. $a_8 - a_9$
3. $a_{28} + a_{29} + a_{30} - 1$
4. $a_{22} + 2a_{24} - 1$
5. $a_{23} - a_{24}$
6. $a_{31} + 2a_{33} - 1$
7. $a_{32} - a_{33}$
8. $a_4 + 2a_6 - 1$
9. $a_5 - a_6$
10. $a_{19} + 2a_{21} - 1$
11. $a_{20} - a_{21}$
12. $a_{25} + 2a_{27} - 1$
13. $a_{26} - a_{27}$
14. $a_1 + 2a_3 - 1$
15. $a_2 - a_3$
16. $3a_8 * a_{30} + a_8 - a_{23} - a_{32}$
17. $a_{23} * a_{29} + 2a_{23} * a_{30} + a_{29} * a_{32} - a_{30} * a_{32} - a_{32}$
18. $3a_8 * a_{29} - 2a_8 + 2a_{23} - a_{32}$
19. $a_5 * a_{29} + 2a_5 * a_{30} - a_5 + a_{20} - a_{26}$
20. $a_2 * a_{29} + 2a_2 * a_{30} - 3a_5 * a_{32}$
21. $3a_5 * a_{23} - 3a_2 * a_{30} + 3a_5 * a_{32} - a_2$
22. $3a_8 * a_{20} - 3a_8 * a_{26} + 3a_5 * a_{32} - a_2$
23. $3a_5 * a_8 - a_2$

- $N = 2221$ (subset)

1. $a_{13} + 2a_{15} - 1$
2. $a_{14} - a_{15}$
3. $a_{28} + a_{29} + a_{30} - 1$
4. $a_{34} + 2a_{36} - 1$
5. $a_{35} - a_{36}$
6. $a_{37} + 2a_{39} - 1$
7. $a_{38} - a_{39}$
8. $a_{10} + 2a_{12} - 1$
9. $a_{11} - a_{12}$
10. $a_4 + 2a_6 - 1$
11. $a_5 - a_6$
12. $a_{19} + 2a_{21} - 1$
13. $a_{20} - a_{21}$
14. $a_{25} + 2a_{27} - 1$
15. $a_{26} - a_{27}$

16. $a_{14} * a_{29} - a_{14} * a_{30} + a_{35} - a_{38}$
17. $a_{11} * a_{29} - a_{11} * a_{30} + 3a_5 * a_{35} - 3a_5 * a_{38}$
18. $a_5 * a_{29} + 2a_5 * a_{30} - a_5 + a_{20} - a_{26}$
19. $3a_{14}*a_{20}-3a_{14}*a_{26}+3a_{11}*a_{30}-3a_5*a_{35}+3a_5*a_{38}-a_{11}$
20. $3a_5 * a_{14} - a_{11}$
21. $a_{20} * a_{29} * a_{35} + 2a_{20} * a_{30} * a_{35} - a_{26} * a_{29} * a_{38} + a_{26} * a_{30} * a_{38} - a_{14} * a_{26} + a_{11} * a_{30} - a_5 * a_{35} - a_{20} * a_{35} - a_{26} * a_{35} + 2a_{20} * a_{38}$

- $N = 2222$ (subset)

1. $a_{16} + 2a_{18} - 1$
2. $a_{17} - a_{18}$
3. $a_{28} + a_{29} + a_{30} - 1$
4. $a_{13} + 2a_{15} - 1$
5. $a_{14} - a_{15}$
6. $a_{31} + 2a_{33} - 1$
7. $a_{32} - a_{33}$
8. $a_7 + 2a_9 - 1$
9. $a_8 - a_9$
10. $a_{34} + 2a_{36} - 1$
11. $a_{35} - a_{36}$
12. $a_{37} + 2a_{39} - 1$
13. $a_{38} - a_{39}$
14. $a_{22} + 2a_{24} - 1$
15. $a_{23} - a_{24}$
16. $a_{10} + 2a_{12} - 1$
17. $a_{11} - a_{12}$
18. $a_4 + 2a_6 - 1$
19. $a_5 - a_6$
20. $a_{19} + 2a_{21} - 1$
21. $a_{20} - a_{21}$
22. $a_{25} + 2a_{27} - 1$
23. $a_{26} - a_{27}$
24. $a_1 + 2a_3 - 1$
25. $a_2 - a_3$
26. $a_{17} * a_{30} - a_{14} * a_{32} - a_8 * a_{35} + a_8 * a_{38}$
27. $3a_8 * a_{30} + a_8 - a_{23} - a_{32}$
28. $a_{23} * a_{29} + 2a_{23} * a_{30} + a_{29} * a_{32} - a_{30} * a_{32} - a_{32}$
29. $a_{17} * a_{29} - a_{14} * a_{32} + 2a_8 * a_{35} - 2a_8 * a_{38}$
30. $a_{14} * a_{29} - a_{14} * a_{30} + a_{35} - a_{38}$
31. $a_{11} * a_{29} - a_{11} * a_{30} + 3a_5 * a_{35} - 3a_5 * a_{38}$
32. $3a_8 * a_{29} - 2a_8 + 2a_{23} - a_{32}$
33. $a_5 * a_{29} + 2a_5 * a_{30} - a_5 + a_{20} - a_{26}$
34. $a_2 * a_{29} + 2a_2 * a_{30} - 3a_5 * a_{32}$
35. $3a_{14} * a_{23} - 3a_8 * a_{35} + 3a_8 * a_{38} - a_{17}$
36. $3a_5 * a_{23} - 3a_2 * a_{30} + 3a_5 * a_{32} - a_2$
37. $a_{17}*a_{20}-a_{11}*a_{23}-a_{17}*a_{26}+a_{11}*a_{32}+a_2*a_{35}-a_2*a_{38}$
38. $3a_{14}*a_{20}-3a_{14}*a_{26}+3a_{11}*a_{30}-3a_5*a_{35}+3a_5*a_{38}-a_{11}$
39. $3a_8 * a_{20} - 3a_8 * a_{26} + 3a_5 * a_{32} - a_2$
40. $a_5 * a_{17} - a_{11} * a_{23} + a_2 * a_{35} - a_2 * a_{38}$
41. $3a_8 * a_{14} - a_{17}$
42. $3a_5 * a_{14} - a_{11}$
43. $a_2 * a_{14} - a_{11} * a_{23} + a_2 * a_{35} - a_2 * a_{38}$
44. $a_8 * a_{11} - a_{11} * a_{23} + a_2 * a_{35} - a_2 * a_{38}$
45. $3a_5 * a_8 - a_2$
46. $a_{20} * a_{29} * a_{35} + 2a_{20} * a_{30} * a_{35} - a_{26} * a_{29} * a_{38} + a_{26} * a_{30} * a_{38} - a_{14} * a_{26} + a_{11} * a_{30} - a_5 * a_{35} - a_{20} * a_{35} - a_{26} * a_{35} + 2a_{20} * a_{38}$
47. $6a_8 * a_{26} * a_{35} - 3a_5 * a_{32} * a_{35} - 3a_{20} * a_{32} * a_{35} - 3a_8 * a_{26} * a_{38} - 3a_{23} * a_{26} * a_{38} + 6a_5 * a_{32} * a_{38} + a_{17} * a_{26} - a_{11} * a_{32} + a_2 * a_{35} - a_2 * a_{38}$

## Algorithms

**Algorithm 1** Inference of a 4-node hybridization cycle in an $n$-taxon phylogenetic network ($n > 8$) with phylogenetic invariants

Table of estimated concordance factors; optional: the number of optimal networks ($m$) to return (default $m = 5$) Top $m$ optimal networks with smallest invariant score scores ← an empty array
subnets ← an empty array
Forfor
**for** $P_i$, a subset of 8 taxa **do do** scores$_i$,subnets$_i$ ← Algorithm in main text (CF, $P_i$, m=2520) Note total number of subnets (partitions for 8 taxa) = 2520
append(scores,scores$_i$)
append(subnets, subnets$_i$) **end**
subnets_sorted ← sort subnets in descending order according to its score
result ← an empty array
  **for** i in 1:length(subnets_sorted) **do do** miss_taxa ← array of taxa that are missing from total $n$ taxa in subnets_sorted[i]
    **for** t in miss_taxa **do do**
      **for** j in (i+1):length(subnets_sorted) **do do**
        **if** t in $n_0$ of subnets_sorted[j] **then** add t to $n_0$ of subnets_sorted[i]
break

Fna
t in $n_3$ of subnets_sorted[j] add t to $n_3$ of subnets_sorted[i]
break
t in $n_1$ of subnets_sorted[j]
**if** ($n_2$ of subnets_sorted[j] == $n_1$ of subnets_sorted[i]) & ($n_1$ of subnets_sorted[j] without t is in $n_2$ of subnets_sorted[i]) **then** add t to $n_2$ of subnets_sorted[i]
break
**else** add t to $n_1$ of subnets_sorted[i]
break
t in $n_2$ of subnets_sorted[j]
**if** ($n_1$ of subnets_sorted[j] == $n_2$ of subnets_sorted[i]) & ($n_2$ of subnets_sorted[j] without t is in $n_1$ of subnets_sorted[i]) **then** add t to $n_1$ of subnets_sorted[i]
break
**else** add t to $n_2$ of subnets_sorted[i] break **end**
**end**
**if** subnets_sorted[i] not in result **then** append(result, subnets_sorted[i])
**if** length(result)==m **then** break **end**
**return** result

## Simulation study

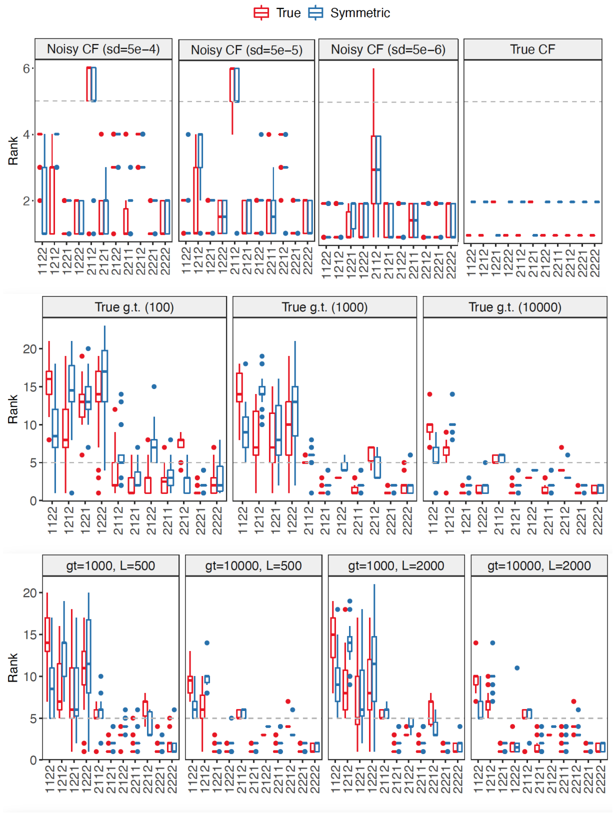


**Fig. 1.** Rank (y-axis) in the invariant score for each network (x-axis) for the true network (red) and its symmetric network (inverted clades $n_1$ and $n_2$, in blue) on 3 different cases. Dashed line corresponds to rank 5. **First row**: true and Gaussian-perturbed CFs. Each panel corresponds to a type of simulation: using true concordance factors (left) and using concordance factors with added Gaussian noise (with increasing standard deviation for noise from left to right). Both the true and symmetric networks are within the top 5 ranked networks by the method in all cases, and are thus, easy to distinguish from wrong networks. **Second row**: true simulated gene trees ("g.t"). Each panel corresponds to a number of simulated gene trees from 100 (left) to 10,000 (right). Both the true and symmetric networks are within the top 5 ranked networks by the method as the number of genes increases, and are thus, easy to distinguish from wrong networks. Only the networks that have one taxon below the hybrid node (1122, 1212, 1221, 1222) do not allow accurate reconstruction which brings into attention the importance of taxon sampling for this method. **Third row**:estimated gene trees. Each panel corresponds to a number of gene trees (g.t. from 1000 to 10,000) and sequence length ($L$ from 500bp to 2000bp). Only the networks that have one taxon below the hybrid node (1122, 1212, 1221, 1222) do not allow accurate reconstruction which brings into attention the importance of taxon sampling for this method.

| **Method** | **Time (seconds)** |
| --- | --- |
| Phylogenetic invariants (our method) | 7.17 |
| `SNaQ` | 80.23 |
| `PhyloNet ML` | 84.34 |
| `PhyloNet MPL` | 17.99 |

**Table 2.** Running times (in seconds) on the inference of network $N = 2222$ from 100 true simulated gene trees by four network methods: 1) our phylogenetic invariants (top row); 2) `SNaQ` [1]; 3) `PhyloNet ML` [3], and 4) `PhyloNet MPL` [4].

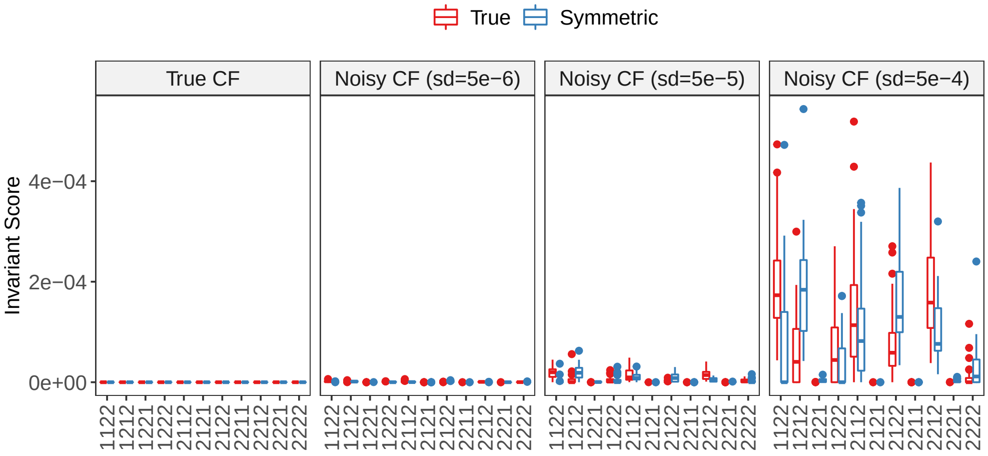


**Fig. 2.** Invariant score (y-axis) measured as $L_2$-norm of the phylogenetic invariants for each network (x-axis) for the true network (red) and its symmetric network (inverted clades $n_1$ and $n_2$, in blue) on the cases of true and Gaussian-perturbed CFs. Each panel corresponds to a type of simulation: using true concordance factors (left) and using concordance factors with added Gaussian noise (with increasing standard deviation for noise from left to right). Both the true and symmetric networks have invariant score close to zero, and are thus, easy to distinguish from wrong networks (whose invariant score is far from zero). As the noise increases, the invariant scores moves away from zero, but still within $10^{-4}$.

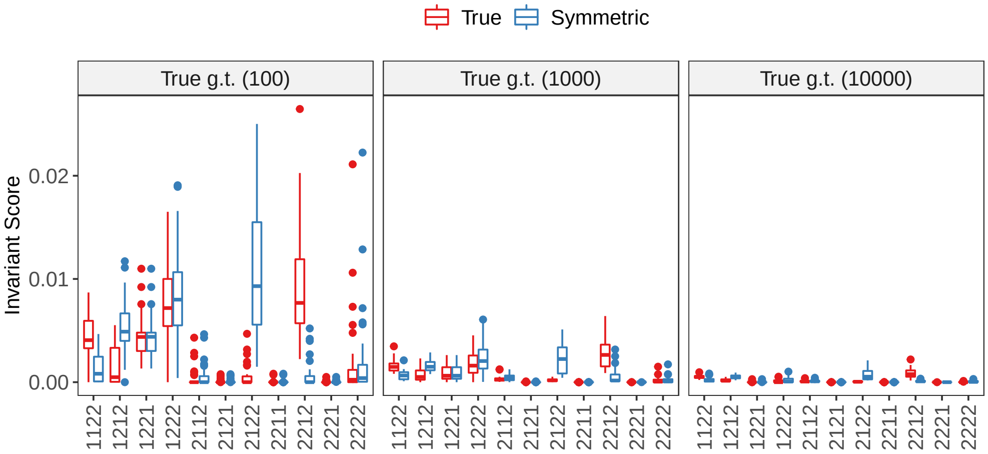


**Fig. 3.** Invariant score (y-axis) measured as $L_2$-norm of the phylogenetic invariants for each network (x-axis) for the true network (red) and its symmetric network (inverted clades $n_1$ and $n_2$, in blue) for the case of true simulated gene trees ("g.t."). Each panel corresponds to a number of simulated gene trees from 100 (left) to 10,000 (right). With true and perturbed concordance factors (top left and top right, respectively). As the number of gene trees increases, the invariant scores of the true and symmetric networks converge to zero, and they are thus, easy to distinguish from wrong networks (whose invariant score is far from zero).

## References

1. Claudia Solís-Lemus and Cécile Ané. "inferring phylogenetic networks with maximum pseudolikelihood under incomplete lineage sorting". *PLoS Genet.*, 12(3):e1005896, March 2016.
2. Claudia R. Solís-Lemus, Arrigo Coen, and Cécile Ané. On the Identifiability of Phylogenetic Networks under a Pseudolikelihood model. *arXiv preprint arXiv:2010.01758*, 2020.
3. Yun Yu et al. Maximum Likelihood Inference of Reticulate Evolutionary Histories. *PNAS*, 111(46):16448–16453, 2014.
4. Yun Yu and Luay Nakhleh. A maximum pseudo-likelihood approach for phylogenetic networks. *BMC Genomics*, 16(10):1–10, 2015.

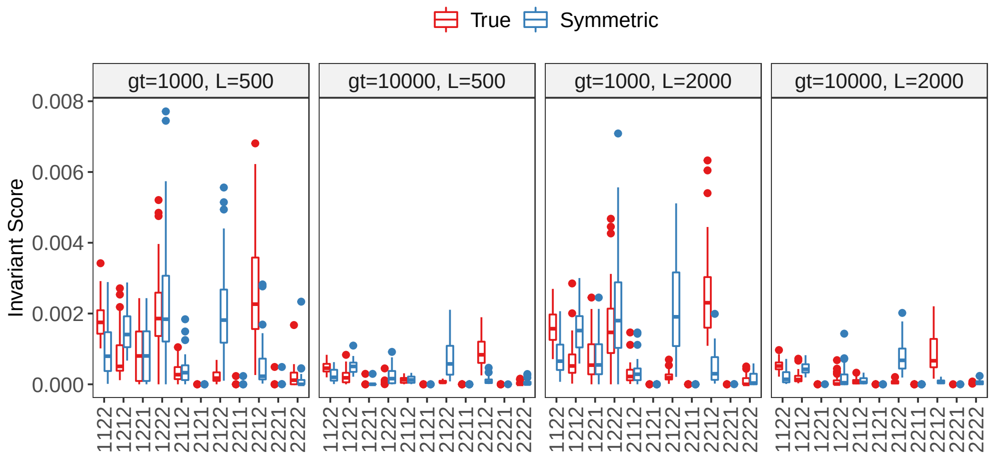


**Fig. 4.** Invariant score (y-axis) measured as $L_2$-norm of the phylogenetic invariants for each network (x-axis) for the true network (red) and its symmetric network (inverted clades $n_1$ and $n_2$, in blue) for the case of estimated gene trees. Each panel corresponds to a number of gene trees (g.t. from 1000 to 10,000) and sequence length ($L$ from 500 to 2000). As the number of gene trees increases, the invariant scores of the true and symmetric networks converge to zero, and they are thus, easy to distinguish from wrong networks (whose invariant score is far from zero).

| Network | True CF | Noisy CF |
|---|---|---|
| 2223 | 1 | 1 |
| 2232 | 1 | 1 (symmetric) |
| 2322 | 1 | 2 |
| 3222 | 1 | 1 |
| 2233 | 1 | 1 (symmetric) |
| 2323 | 1 | 2 (symmetric) |
| 3223 | 1 | 1 |
| 2332 | 1 | 2 (symmetric) |
| 3232 | 1 | 2 (symmetric) |
| 3322 | 1 | 1 (symmetric) |

**Table 3.** Rank of the true (or symmetric) network under the two types of simulations: true CFs (left column) and Gaussian-perturbed CFs (right column) for a level of noise of $\sigma = 0.0005$. In all cases, either the true network or the symmetric networks are within the top 2 of networks identified by the method which proves that our algorithm for more than 8 taxa that builds on Algorithm **??** works appropriately.
